# Single‐Cell Map of Early Lung Adenocarcinoma Uncovers Invasion‐Associated Tertiary Lymphoid Structures

**DOI:** 10.1002/advs.78670

**Published:** 2026-11-09

**Authors:** Pengfei Ma, Chunming He, Mei‐Chun Cai, Jie Wu, Haijiao Lu, Shichao Zhou, Zhenfeng Zhang, Qing Zhang, Yunqing Chen, Jingyu Zang, Kaiyan Ye, Yu Shu, Chee Wai Chua, Hui Li, Yun Fan, Jing Wang, Zhuang Yu, Xinying Xue, Xiaojing Zhao, Guanglei Zhuang, Yujie Fu

**Affiliations:** ^1^ State Key Laboratory of Systems Medicine For Cancer Department of Thoracic Surgery Shanghai Cancer Institute Ren Ji Hospital Shanghai Jiao Tong University School of Medicine Shanghai China; ^2^ Department of Obstetrics and Gynecology Affiliated Hospital of Zunyi Medical University Zunyi China; ^3^ Department of Pathology The Affiliated Hospital of Qingdao University Qingdao China; ^4^ Department of Respiratory and Critical Care Medicine Shanghai Chest Hospital Shanghai Jiao Tong University School of Medicine Shanghai China; ^5^ Department of Thoracic Medical Oncology Hangzhou Institute of Medicine (HIM) Zhejiang Cancer Hospital Chinese Academy of Sciences Hangzhou China; ^6^ State Key Laboratory of Systems Medicine For Cancer Shanghai Cancer Institute Department of Urology Ren Ji Hospital Shanghai Jiao Tong University School of Medicine Shanghai China; ^7^ Department of Oncology The Affiliated Hospital of Qingdao University Qingdao China; ^8^ Department of Respiratory and Critical Care National Clinical Research Center for Geriatric Diseases Xuanwu Hospital of Capital Medical University Beijing China; ^9^ Department of Respiratory and Critical Care Shandong Second Medical University Shandong China; ^10^ Shanghai Key Laboratory of Gynecologic Oncology Ren Ji Hospital Shanghai Jiao Tong University School of Medicine Shanghai China

**Keywords:** adenocarcinoma, biology, cancer research, immune system, immunohistochemistry, lung, pathology

## Abstract

Early lung adenocarcinoma presenting as ground‐glass nodules (GGNs) is increasingly detected in clinical practice. However, robust biomarkers reflecting progression from in situ to invasive disease are incompletely defined. Here, we integrated single‐cell RNA sequencing, T‐cell receptor repertoire profiling, whole‐slide immunohistochemistry, multiplex immunohistochemistry, and an orthotopic murine lung adenocarcinoma model to dissect the immune landscape of primary GGNs. We found that these early thoracic lesions harbored abundant infiltrates of innate and adaptive immune cells, but intratumoral T lymphocytes included putative polyclonal bystander clones embedded in an immunosuppressive microenvironment. We further observed a marked stage‐dependent increase in T and B cell‐rich tertiary lymphoid structures (TLSs), dominated by immature configurations, whereas mature TLSs remained relatively infrequent. In mouse allografts, spontaneously arising TLSs were likewise underdeveloped, and LTαβ or LIGHT overexpression, as well as PD‐1 blockade, promoted TLS neogenesis and maturation, accompanied by reduced tumor growth. Collectively, our study delineates the immunologic features associated with pathological invasion, identifies a predominance of structurally immature TLS niches, and provides a rationale for further investigating the therapeutic potential of promoting TLS maturation to intercept early lung adenocarcinoma.

## Graphical abstract

Early thoracic lesions are already heavily infiltrated by immune cells, yet these infiltrates largely consist of bystander T lymphocytes within an immunosuppressive microenvironment. Pathological invasion is strikingly associated with the neogenesis of T/B cell‐rich TLSs that predominantly remain immature. LTβR ligand overexpression or PD‐1 blockade promotes TLS neogenesis and maturation while constraining allograft growth, suggesting a link between TLS developmental state and tumor progression in early lung adenocarcinoma.

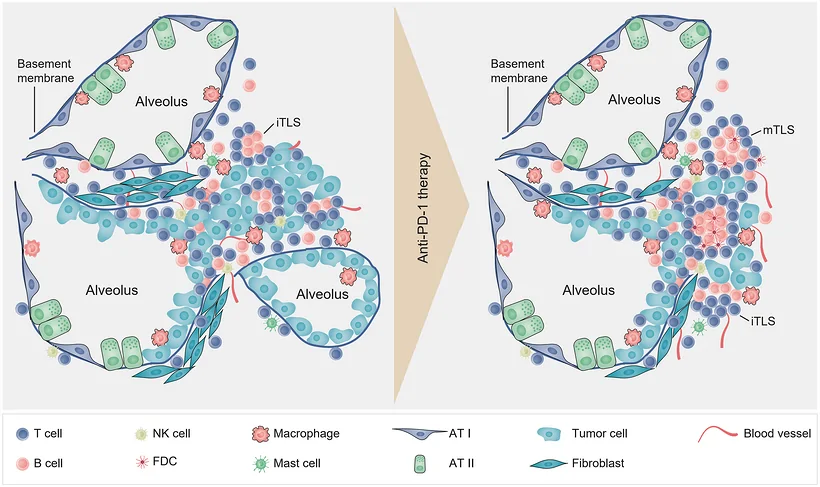

## 1 Introduction

Lung cancer is the most lethal malignancy worldwide [1], and early diagnosis is key to successful management [2, 3]. Adenocarcinoma is the major histotype [4], which at the initial stage often presents as pulmonary ground‐glass nodules (GGNs) on computed tomography (CT) images. These GGNs are radiologically classified into two general categories, i.e., pure GGNs (pGGNs) with only ground‐glass components indicating lepidic growth, and mixed GGNs (mGGNs) that also contain a consolidated region corresponding to tumor invasion [5]. However, the correlation between radiographic manifestation and pathological examination is not absolute [6, 7]. As a result, current risk assessment and preoperative decision‐making remain fairly empirical and can be challenging due to heterogeneous disease behaviors [8, 9, 10]. Therefore, it is crucial to gain an improved understanding of the esoteric biology underpinning early lung carcinogenesis. In particular, the multicellular makeup of GGNs merits elucidation to aid biomarker identification and guideline development beyond physician experience or patient preference.

The majority of neoplastic GGNs are diagnosed as thoracic lesions belonging to a histopathological continuum from atypical adenomatous hyperplasia (AAH), through adenocarcinoma in situ (AIS), followed by minimally invasive adenocarcinoma (MIA), and eventually to fully invasive adenocarcinoma (IAC). During these consecutive progression steps, in situ to invasive transition represents a critical point in the evolutionary trajectories of lung adenocarcinoma, leading to an unfavorable prognosis and more aggressive treatment recommendations [11, 12, 13, 14, 15, 16]. Recent genomic efforts have focused on tumor‐intrinsic factors and highlighted potentially causative somatic aberrations driving cancer outgrowth [6, 17, 18, 19, 20, 21, 22, 23, 24, 25, 26]. Single‐cell studies have also characterized the multicellular composition of subsolid lung nodules [27, 28]. More recently, spatial analyses have delineated immune organization across lung adenocarcinoma progression and identified inflammatory niches associated with alveolar progenitors [29, 30]. Meanwhile, when precursor cells penetrate across the basement membrane, they acquire access to interstitial tissues and intermingle with extra‐alveolar parenchyma at the interface. Nevertheless, the cellular architecture of invading tumor front and its spatiotemporal interactions with the surrounding microenvironment remain ambiguous.

Here, by integrating unbiased single‐cell RNA sequencing (scRNA‐seq), T‐cell repertoire profiling, whole‐slide immunohistochemistry (IHC), and multiplex IHC (mIHC), we demonstrated pervasive immune infiltration in a unique cohort of primary ground‐glass nodules including pGGNs and mGGNs. We observed an increased abundance of T and B cell‐rich immature tertiary lymphoid structures in association with pathological invasion, alongside putative bystander lymphocyte infiltration and immune suppression. In an orthotopic murine allograft model, LTβR ligand overexpression or PD‐1 blockade increased TLS density and structural maturation, accompanied by reduced tumor growth. These findings support further investigation of therapeutic TLS modulation in early lung adenocarcinoma.

## 2 Results and Discussion

### 2.1 The Single‐Cell Landscape of human GGNs

To address the knowledge gap in early lung cancer pathogenesis, we undertook a systematic approach to characterize the cellular and molecular landscape of treatment‐naïve adenocarcinoma presenting as GGNs (Figure 1A; Figure S1). Initially, droplet‐based scRNA‐seq was employed to resolve diverse cell compositions of 12 radiologically diagnosed and pathologically confirmed neoplastic specimens including 1 AIS, 5 MIA, and 6 IAC (Figure S2A; Table S1). A total of 46,377 single‐cell profiles passed rigorous quality control, yielding 19 stable clusters independent of patient sources (Figure S2B; Table S2). We manually curated cell subsets into eight major lineages on the basis of canonical markers (Figure 1B): T cell, B cell, myeloid cell, NK (natural killer) cell, epithelial cell, endothelial cell, mast cell, and fibroblast (Figure 1C; Figure S2C; Table S3). Signature genes were identified for all cell types (Figure S3A; Table S4) and specifically distinguished each compartment from the others (Figure S3B). These data defined the single‐cell landscape of heterogeneous human pulmonary GGNs at high resolution.

**FIGURE 1:**
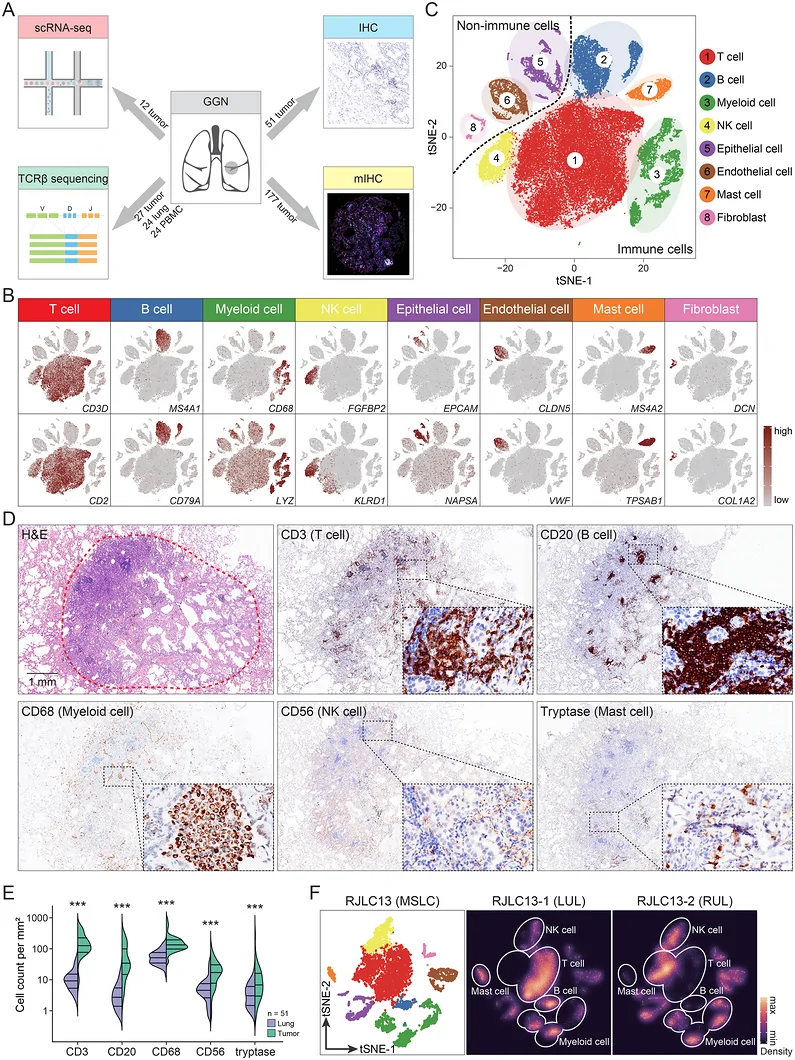
The single‐cell landscape of human lung GGNs. (A) Schematic overview of the experimental design. Human lung GGNs were subjected to scRNA‐seq (n = 12), TCRβ‐seq (n = 27), IHC staining (n = 51), and mIHC analysis (n = 177). (B) t‐SNE plots showing gene expression of selected markers for the eight major cell types identified by scRNA‐seq analysis. (C) t‐SNE plot showing the eight major cell types identified by scRNA‐seq analysis. Each dot corresponds to a single cell colored by cell type. (D) Representative images of H&E and IHC staining for CD3 (T cell), CD20 (B cell), CD68 (myeloid cell), CD56 (NK cell), and tryptase (mast cell) in a cohort of 51 human lung GGNs. Scale bar, 1 mm. (E) Quantification of CD3+, CD20+, CD68+, CD56+, and tryptase+ cell densities in tumor lesions and normal tissues from a cohort of 51 human lung GGNs. ∗∗∗p < 0.001, two‐sided paired t test with Holm correction. (F) t‐SNE plots illustrating different cell compositions and gene expression profiles from two nodules of an MSLC patient (RJLC13).

A notable observation was the overwhelming innate (myeloid, NK, and mast cell) and adaptive (T and B cell) immune infiltrates across early neoplasms, in keeping with a recent single‐cell analysis of subsolid nodules [27]. IHC staining for CD3, CD20, CD68, CD56, and tryptase in an expanded GGN collection (n = 51; Table S5) corroborated the scRNA‐seq results (Figure 1D), and displayed increased densities of positive cells in tumor regions compared to their normal counterparts (CD3: mean difference = 157.92 cells/mm^2^, 95% CI 124.95–195.77, p < 0.001; CD20: mean difference = 63.90 cells/mm^2^, 95% CI 44.76–84.87, p < 0.001; CD68: mean difference = 116.91 cells/mm^2^, 95% CI 82.69–159.66, p < 0.001; CD56: mean difference = 15.97 cells/mm^2^, 95% CI 10.69–21.90, p < 0.001; tryptase: mean difference = 11.32 cells/mm^2^, 95% CI 4.36–22.94, p < 0.001) (Figure 1E). Therefore, the elevated opacities on CT scans could be in part ascribed to leukocyte aggregates aside from epithelial hyperplasia and local fibrosis.

Despite overall similarity, substantial inter‐lesion heterogeneity of the immune milieu was consistently captured by both scRNA‐seq (Figure S4A) and IHC (Figure S4B). This phenotype was best illustrated in a multiple synchronous lung cancer (MSLC) case (RJLC13), exhibiting not only private cellular structure but also differential gene expression within the two nodules in contralateral lobes (Figure 1F). To further gauge immune cell configuration, we analyzed pairwise correlation of individual proportions across lesions and found evident co‐infiltration of T and B lymphocytes (Figure S4C). The significant T and B cell co‐abundance pattern was recapitulated in the IHC cohort (Figure S4D) and remained evident across solid tumors at the pan‐cancer scale (Figure S4E). Of particular interest, T and B cell gene signatures derived from the scRNA‐seq assay stratified TCGA lung adenocarcinomas into two molecular subtypes (Figure S5A) showing distinct clinicopathological features (Figure S5B), and correlated with reported response rates to immunotherapy across cancer types (Figure S5C). Collectively, we proposed that early lung adenocarcinoma manifesting as GGNs was hallmarked by complex immune constituents involving a prominent T and B cell interplay with potential clinical relevance.

### 2.2 Tumor Infiltration of Bystander Lymphocytes

We sought to thoroughly characterize tumor‐infiltrating immune cells in GGNs. A core transcriptional program (*CREM*, *RGS2*, *SLA*, *NFE2L2*) of human lung‐resident leukocytes suggested plausibly multifarious cellular origins (Figure 2A). Focusing on the well‐studied T lymphocytes (Figure 2B), we assembled a panel of putative resident (*CCR5*, *CXCR6*, *ITGA1*) and circulating (*CCR7*, *SELL*, *S1PR1*) markers as coherent classifiers to identify resident‐like (R3+C3‐) and circulating‐like (R3‐C3+) T‐cell subsets, which respectively expressed representative trafficking molecules (Figure 2C; Table S6). Although each subject contained variable fractions of R3+C3‐ and R3‐C3+ T cells (Figure 2D), our results, in accordance with the emerging model of immune surveillance, implicated both locoregional and recirculating lymphocytes in early stages of lung tumorigenesis.

**FIGURE 2:**
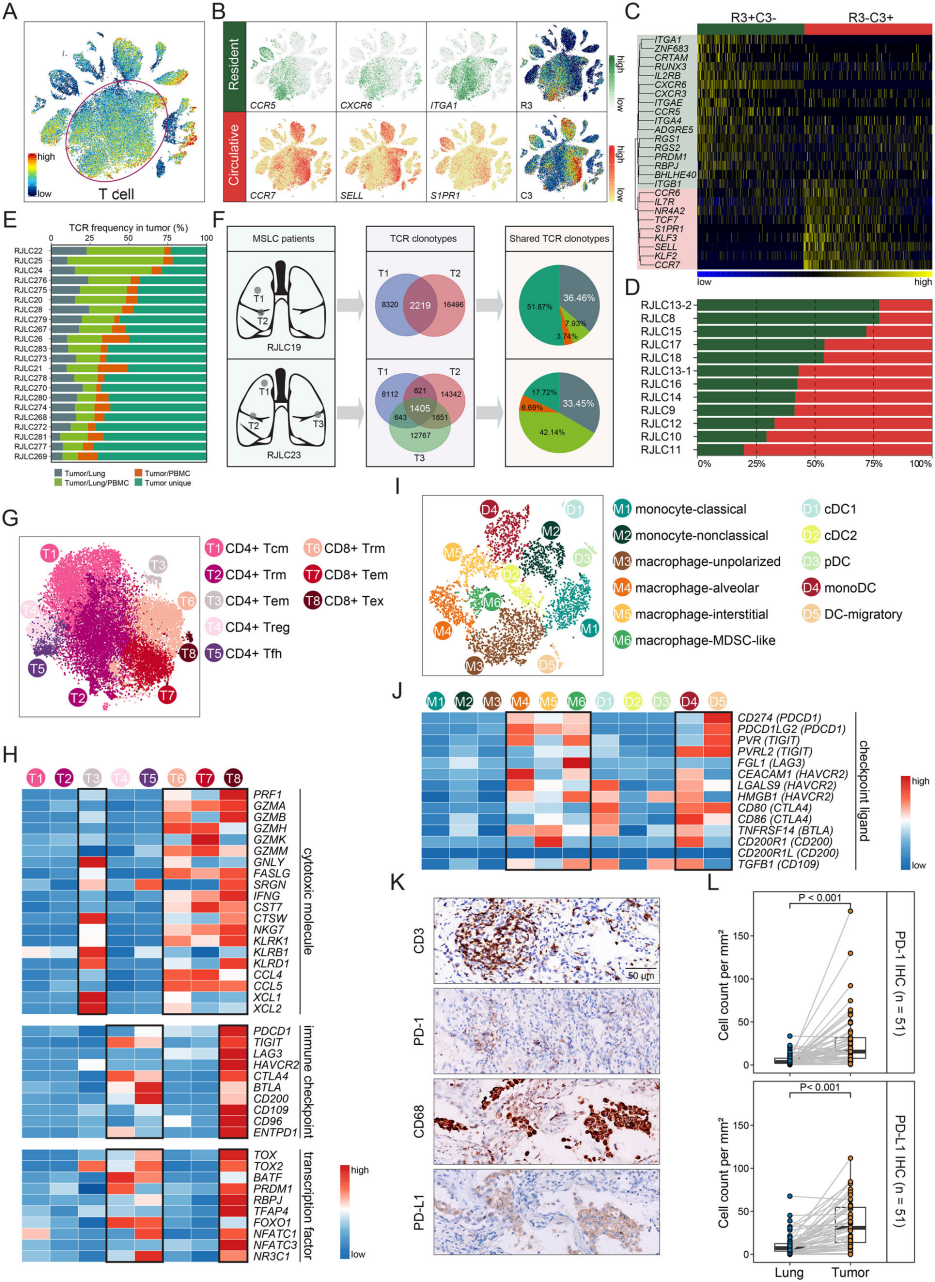
Tumor infiltration of bystander lymphocytes and immune suppression. (A) t‐SNE plot showing lung‐resident signature scores across all single cells. The red circle highlights T cells. (B) t‐SNE plots showing expression of resident T cell marker genes (top) and circulating T cell marker genes (bottom) across all single cells. Composite signature scores for resident T cells (R3) and circulating T cells (C3) are shown at right. (C) Heatmap of 17 resident and 9 circulating T cell‐related genes comparing resident (R3+C3−) and circulating (R3−C3+) T cell populations. (D) Quantification of resident (green) and circulating (red) T cells in each human lung GGN. (E) TCR frequencies in 22 patients with lung GGNs as categorized into four groups: Tumor unique (only detected in tumor lesions), Tumor/Lung (shared with matched lung tissues), Tumor/PBMC (shared with matched PBMC), and Tumor/Lung/PBMC (shared across all matched samples). (F) Venn diagram showing shared TCR clonotypes in lung GGNs of two MSLC patients (RJLC19 and RJLC23). The pie chart depicts the distribution of shared TCR clonotypes in four categories (Tumor unique, Tumor/Lung, Tumor/PBMC, Tumor/Lung/PBMC). (G) t‐SNE plot showing eight clusters of 25,152 T cells identified by scRNA‐seq analysis from 12 human lung GGNs. Each dot is a single cell colored by cluster. (H) Heatmap showing expression of selected key genes related to T cell function across the eight T‐cell clusters. (I) t‐SNE plot showing eleven clusters of 5,075 myeloid cells identified by scRNA‐seq analysis from 12 human lung GGNs. Each dot is a single cell colored by cluster. (J) Heatmap showing expression of selected checkpoint ligands across the eleven myeloid cell clusters. (K) Representative images of IHC staining for CD3 (T cell), PD‐1, CD68 (myeloid cell), and PD‐L1 in a cohort of 51 human lung GGNs. Scale bar, 50 µm. (L) Quantification of PD‐1+ and PD‐L1+ cell densities in tumor lesions and paired normal tissues from a cohort of 51 human lung GGNs. P values were calculated using two‐sided paired t test.

To support the above notion, the CDR3 region of the T‐cell receptor (TCR) β chain was sequenced in matched peripheral blood mononuclear cells (PBMC), non‐tumor lung tissue, and tumor specimens from 22 patients with solitary GGNs (Table S7). All samples contained a large number of productive TCR sequences (PBMC: 5,645,148 ± 1,143,816, mean ± s.d.; lung: 3,445,586 ± 997,489, mean ± s.d.; tumor: 4,757,021 ± 2,670,892, mean ± s.d.) and unique TCR clonotypes (PBMC: 32,229 ± 22,950, mean ± s.d.; lung: 7,645 ± 4,806, mean ± s.d.; tumor: 14,014 ± 9,642, mean ± s.d.), reflecting polyclonal T‐cell repertoires (Figure S6A). TCR diversities of PBMC, lung, and tumor samples were significantly different, as assessed by normalized Shannon entropy (PBMC: 0.78 ± 0.06, mean ± s.d.; lung: 0.71 ± 0.06, mean ± s.d.; tumor: 0.76 ± 0.07, mean ± s.d.) and clonality index (PBMC: 0.22 ± 0.06, mean ± s.d.; lung: 0.29 ± 0.06, mean ± s.d.; tumor: 0.24 ± 0.07, mean ± s.d.) (Figure S6B). Notably, the Jaccard and Horn indices both indicated greater TCR repertoire similarity between tumor and lung than between either tissue and PBMC (Figure S6C). Consistent with this pattern, relatively more T lymphocytes in GGNs appeared to originate from host lungs (36.9% ± 16.2%, mean ± s.d.) than from PBMCs (29.8% ± 14.6%, mean ± s.d.) (Figure 2E). Using TCR sequencing in two additional patients with MSLCs as exceptional examples (Table S7), we observed that physically separated nodules shared numerous TCR clonotypes, a considerable portion of which were also detected in matched lung tissue and PBMC (Figure 2F).

An important question concerned the neoantigen reactivity of intratumoral T cells. We found that the abundance distribution of TCR repertoires approximately fit power‐law models with comparable estimated exponents in PBMC (1.30 ± 0.08, mean ± s.d.), lung (1.33 ± 0.06, mean ± s.d.), and tumor (1.32 ± 0.07, mean ± s.d.) (Figure S6D), implying the long‐tailed fractal structure and continuously itinerant property of T lymphocytes. Identical rules were unveiled by recent publications and in line with the widespread presence of cancer‐unrelated bystanders [31, 32, 33]. Hence, we retained only clonally expanded TCRs for downstream analysis, using a frequency threshold of > 0.001 to enroll approximately the top 1% of ranked clonotypes (Figure S7A), which collectively comprised more than 30% of productive TCR sequences (Figure S7B). Even though expanded TCR profiles differed markedly among patients (Figure S8A), they were preferentially associated with common microbial pathogens such as Epstein‐Barr virus, cytomegalovirus, and influenza virus (Figure S8B). The inferred viral TCR sequences also at least partially accounted for the ubiquitous T‐cell repertoire in PBMC, lung, and tumor samples (Figure S8C). Taken together, these findings suggested that human pulmonary GGNs were populated by tumor‐ignorant immune infiltrates pre‐existing at mucosal site or in peripheral blood.

### 2.3 Evidence for Immune Suppression

To further evaluate the functional states of lymphocytic infiltrates in GGNs, we performed unsupervised graph‐based clustering of 25,152 T cells and identified eight distinct subclusters comprising five CD4+ and three CD8+ T‐cell subsets (Figure 2G) based on canonical marker expression (Figure S9A) cross‐referenced with established gene signatures from the literature [34, 35, 36] (Figure S9B). Although several T‐cell subsets displayed elevated expression of cytotoxic molecules, prominent upregulation of immune checkpoint receptors and immunoinhibitory transcription factors was observed in CD4+ regulatory T cells (CD4+ Treg), CD4+ follicular helper T cells (CD4+ Tfh), and CD8+ exhausted T cells (CD8+ Tex) (Figure 2H), suggesting a functionally impaired status of T‐cell subpopulations. In parallel, tumor‐infiltrating myeloid cells could be divided into 11 different subclusters spanning two monocyte, four macrophage, and five dendritic cell (DC) subsets (Figure 2I; Figure S9C). Remarkably, multiple macrophage and DC subpopulations demonstrated evident gene expression of cognate immune checkpoint ligands (Figure 2J). IHC staining for CD3, PD‐1, CD68, and PD‐L1 in an expanded GGN cohort (n = 51; Table S5) corroborated the transcriptomic findings from scRNA‐seq analysis (Figure 2K), with significantly higher densities of PD‐1 (mean difference = 21.70 cells/mm^2^, 95% CI 13.13–31.63, p < 0.001) and PD‐L1 (mean difference = 24.53 cells/mm^2^, 95% CI 18.12–31.36, p < 0.001) in tumor regions compared to their normal counterparts (Figure 2L). Collectively, these results highlighted the emergence of an immunosuppressive microenvironment despite abundant immune infiltration in early lung adenocarcinoma presenting as GGNs.

### 2.4 Identification of Invasion‐Associated TLSs

Leveraging the scRNA‐seq data, we systematically compared cell composition across histological categories and observed a significantly higher frequency of B cell infiltration in IAC compared with AIS/MIA (median, 16.4% vs. 3.2%; p = 0.017) (Figure 3A). This increase was not obvious when stratified by EGFR mutation status or clinical parameters including smoking history, gender, and age (Figure S10A). IHC confirmed a dramatic accumulation of CD20+ B cells from AIS/MIA to IAC (Figure 3B), which aggregated into distinctive clusters reminiscent of tertiary lymphoid structures (TLSs) [37]. Indeed, T cells marked by CD3, CD4, and CD8 were also present (Figure S10B). We assembled multiple GGN cohorts to validate the relationship between TLS formation and pathological invasion. First, mGGNs harbored a substantially greater TLS burden than pGGNs (median difference = 0.12 TLSs/mm^2^, 95% CI 0.06–0.16, p < 0.001) (Figure 3C; Figure S10C; Table S8). Second, TLS density increased in a stepwise manner across AIS, MIA, and IAC (MIA vs AIS: median difference = 0.09 TLSs/mm^2^, 95% CI 0.03–0.13, p < 0.001; IAC vs MIA: median difference = 0.14 TLSs/mm^2^, 95% CI 0.08–0.19, p < 0.001) (Figure 3D; Figure S10D; Table S9). Third, in 46 patients with MIA, TLS density was significantly higher in invasive regions compared with paired pre‐invasive regions within the same tumor (median difference = 0.23 TLSs/mm^2^, 95% CI 0.10–0.33, p < 0.001) (Figure 3E; Table S10). Fourth, this pattern was further corroborated in an MSLC cohort (n = 17), in which within‐patient comparisons consistently showed higher TLS density in IAC than in AIS/MIA lesions (Figure 3F; Table S11). Therefore, our integrated analyses of TLS distribution, histopathological subtypes, and GGN radiologic features demonstrated that pathological invasion was associated with pronounced TLS neogenesis in early lung adenocarcinoma (Figure 3G).

**FIGURE 3:**
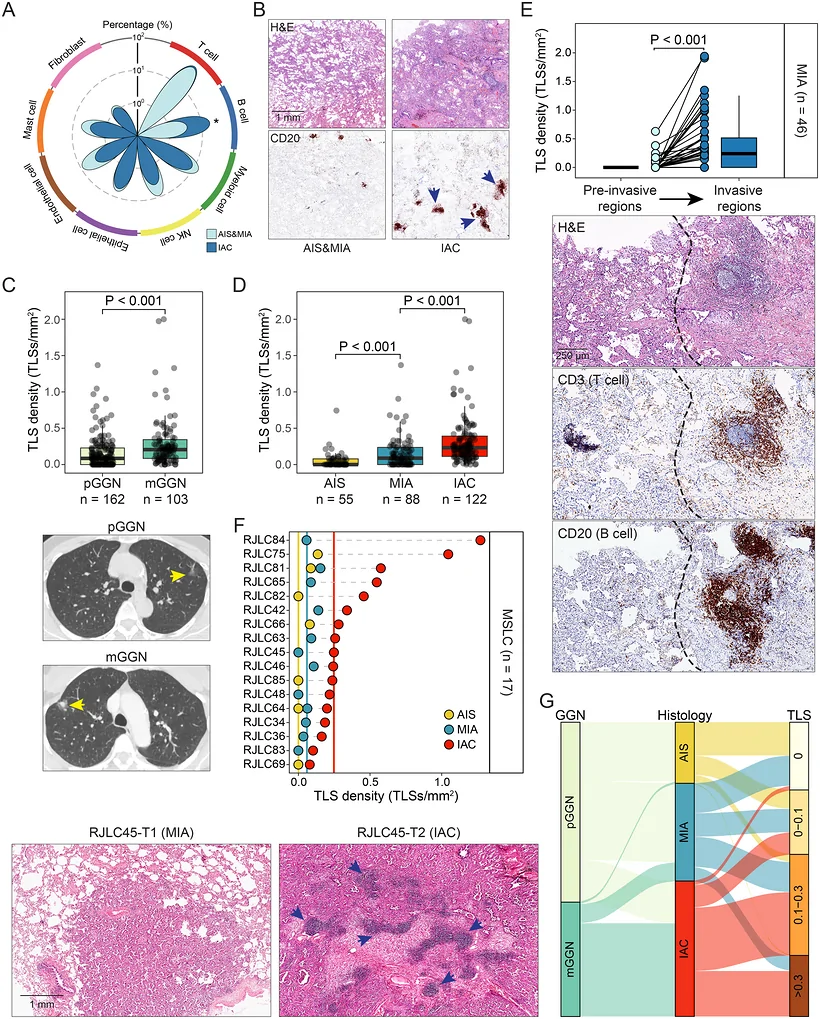
Identification of invasion‐associated TLSs. (A) Radar plot showing the distribution of eight major cell types in human lung GGNs diagnosed with AIS/MIA (n = 6) vs. IAC (n = 6). ∗p < 0.05, two‐sided Mann–Whitney U test with Benjamini–Hochberg adjustment. (B) Representative images of H&E and IHC staining for CD20 (B cell) in human lung GGNs diagnosed with AIS/MIA vs. IAC. Scale bar, 1 mm. (C) Quantification of TLS density in a cohort of 265 human lung GGNs diagnosed with pGGN (n = 162), or mGGN (n = 103). Representative CT images are shown. Yellow arrows indicate GGNs. *p* value was calculated using two‐sided Mann–Whitney U test. (D) Quantification of TLS density in a cohort of 265 human lung GGNs diagnosed with AIS (n = 55), MIA (n = 88), or IAC (n = 122). P values were calculated using two‐sided Mann–Whitney U test with Holm correction. (E) Quantification of TLS density in pre‐invasive and invasive regions from a cohort of 46 human lung GGNs diagnosed with MIA. Representative images of H&E and IHC staining for CD3 (T cell) and CD20 (B cell) are shown. Scale bar, 250 µm. P values were calculated using two‐sided exact sign test after excluding 16 zero differences. (F) Quantification of TLS density across multiple lesions in a cohort of 17 MSLC patients with lung GGNs. H&E staining for an MSLC patient (RJLC45) with MIA and IAC lesions is shown. Blue arrows indicate TLSs. Scale bar, 1 mm. (G) Sankey diagram showing the distribution of human lung GGNs by CT features, pathology, and TLS density.

### 2.5 Modulation of TLS Maturation

The association between TLS formation and tumor invasion was counterintuitive, given that TLSs are considered local hubs sustaining adaptive immune responses and their presence has been linked to improved clinical outcomes in different cancer types [38, 39, 40]. Since TLS function is dependent on its maturation status [41, 42, 43, 44, 45], we performed multiplex IHC (mIHC) in a cohort of 177 GGNs (Figure S11A), comprising 32 AIS, 38 MIA, and 107 IAC (Table S12), to assess TLS developmental states. On the basis of CD21+ follicular dendritic cell network [41, 46, 47], TLSs were classified as immature TLSs (iTLSs) or mature TLSs (mTLSs) (Figure 4A). Consistent with our earlier results, total TLS density was higher in IAC than in AIS or MIA (IAC vs AIS: median difference = 0.59 TLSs/mm^2^, 95% CI 0.40–0.77, p < 0.001; IAC vs MIA: median difference = 0.59 TLSs/mm^2^, 95% CI 0.13–0.76, p = 0.005) (Figure 4B). Interestingly, iTLS density mirrored this increase (IAC vs AIS: median difference = 0.49 TLSs/mm^2^, 95% CI 0.32–0.60, p < 0.001; IAC vs MIA: median difference = 0.49 TLSs/mm^2^, 95% CI 0.01–0.60, p = 0.007) (Figure 4C), whereas mTLS density did not differ significantly across histologic stages (Figure 4D). Thus, the higher TLS density associated with pathological invasion primarily reflected an increase in structurally immature TLSs in early lung adenocarcinoma (Figure 4E).

**FIGURE 4:**
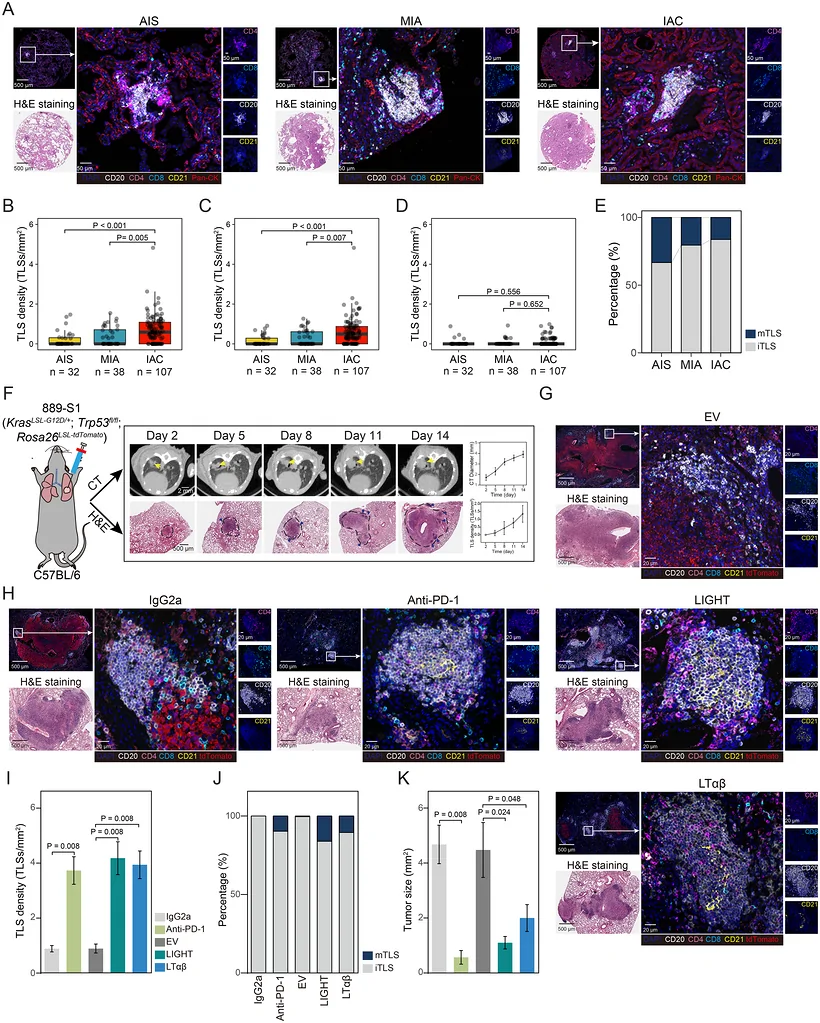
Modulation of TLS maturation. (A) Representative images of mIHC staining of TLSs in AIS (n = 32), MIA (n = 38), and IAC (n = 107) from a cohort of 177 human lung GGNs. (B) Quantification of TLS density in AIS (n = 32), MIA (n = 38), and IAC (n = 107) from a cohort of 177 human lung GGNs. P values were calculated using two‐sided Mann–Whitney U test with Holm correction. (C) Quantification of iTLS density in AIS (n = 32), MIA (n = 38), and IAC (n = 107) from a cohort of 177 human lung GGNs. P values were calculated using two‐sided Mann–Whitney U test with Holm correction. (D) Quantification of mTLS density in AIS (n = 32), MIA (n = 38), and IAC (n = 107) from a cohort of 177 human lung GGNs. P values were calculated using two‐sided Mann–Whitney U test with Holm correction. (E) Stacked bar graph showing the proportions of iTLS and mTLS in AIS (n = 32), MIA (n = 38), and IAC (n = 107) from a cohort of 177 human lung GGNs. (F) Schematic of the orthotopic murine lung adenocarcinoma model. CT and H&E staining were used to evaluate tumor growth and TLS formation. (G) Representative images of mIHC staining of TLSs in the orthotopic murine lung adenocarcinoma model. Three experimental groups were included: EV (empty vector, n = 5), LIGHT overexpression (n = 5), and LTαβ overexpression (n = 5). (H) Representative images of mIHC staining of TLSs in the orthotopic murine lung adenocarcinoma model. Two experimental groups were included: IgG2a isotype control (n = 5) and anti‐PD‐1 treatment (n = 5). (I) Quantification of TLS density in the orthotopic murine lung adenocarcinoma model. P values were calculated using two‐sided exact Mann–Whitney U test with Holm correction. (J) Stacked bar graph showing the proportions of iTLS and mTLS in the orthotopic murine lung adenocarcinoma model. mTLSs were defined by the presence of a discernible CD21‐positive follicular dendritic cell network. (K) Quantification of tumor size in the orthotopic murine lung adenocarcinoma model. P values were calculated using two‐sided exact Mann–Whitney U test with Holm correction.

We reasoned that promoting intratumoral TLS maturation might represent a potential strategy to hinder tumor progression. To test this hypothesis, we established an orthotopic murine lung adenocarcinoma model via intrapulmonary injection of 889‐S1 cells [48]. Allograft burden was monitored by micro‐CT imaging, and TLSs were enumerated on H&E‐stained lung sections (Figure 4F; Figure S11B). In line with our human data, TLS density increased progressively, but most TLSs remained immature in this model. Subsequently, both genetic (Figure 4G) and pharmacologic (Figure 4H) approaches were exploited to facilitate TLS maturation. We engineered 889‐S1 cells to overexpress either LIGHT or LTαβ [49], and confirmed successful transgene expression by qPCR (Figure S11C). In parallel, anti‐PD‐1 treatment was administered to enhance TLS development. Strikingly, LTαβ or LIGHT overexpression, as well as PD‐1 blockade, increased TLS density (Figure 4I; Table S13) and the proportion of mature TLSs (Figure 4J), accompanied by reduced tumor growth (Figure 4K). In summary, these data provided a rationale for further investigating whether therapeutic TLS modulation could intercept early lung adenocarcinoma.

## 3 Discussion

A deeper understanding of the cellular architecture and immunologic remodeling that accompany the evolution of early lung adenocarcinoma manifesting as GGNs is critical for refining risk stratification and therapeutic decision‐making in this increasingly common clinical entity [28, 50, 51]. By leveraging an integrated approach, our study delineates a comprehensive immune atlas of primary GGNs. We show that early thoracic lesions are already heavily infiltrated by immune cells, yet these infiltrates include putative bystander T lymphocytes embedded in an immunosuppressive microenvironment. In parallel, we uncover a striking association between pathological invasion and the neogenesis of T and B cell‐rich TLSs that primarily remain immature. Finally, we demonstrate that LTβR ligand overexpression or PD‐1 blockade is associated with TLS neogenesis and maturation, accompanied by constrained allograft growth, suggesting a possible link between TLS developmental state and tumor progression in the early lung adenocarcinoma setting.

Our data extend prior work showing that immune cells are abundant in subsolid nodules, but their existence alone does not guarantee effective disease control [28, 29, 30, 31, 52, 53, 54]. We observed extensive infiltration by both innate (myeloid, NK, mast) and adaptive (T and B) compartments across AIS, MIA, and IAC, with substantial inter‐lesion variation but a robust pattern of coordinated T and B cell recruitment. TCR repertoire analysis revealed that a substantial fraction of intratumoral T cells shared clonotypes with adjacent lung and peripheral blood, displayed power‐law frequency distributions, and were enriched for viral motifs. These observations are consistent with recent studies indicating that bystander T cells commonly infiltrate lung cancer and can be exploited under appropriate cytokine or immunotherapeutic cues [31, 32, 33]. At the same time, we identified multiple T‐cell subsets bearing exhaustion‐related or inhibitory receptors and myeloid populations expressing cognate checkpoint ligands, collectively supporting the early installation of a suppressive immune niche. Thus, while leukocyte infiltration is a ubiquitous feature of GGNs, the specificity and quality of these infiltrates, as opposed to their sheer abundance, appear to be decisive for whether they constrain or accommodate invasive growth.

Within this immunologically rich environment, we observed a pronounced and stage‐dependent increase of lymphocytic aggregation into TLSs. Our finding complements previous studies of lymphoid organization in lung adenocarcinoma. TLS density has been associated with nodule radiological features [55, 56], while spatial analyses have demonstrated differences in TLS distribution across histological patterns within lung adenocarcinoma [26]. Other studies have characterized TLS organization and associated immune‐cell populations in early lung adenocarcinoma [47, 57] and identified TLS‐like lymphoid aggregates associated with multicellular ecotypes across the disease spectrum [52]. Our study extends these observations through paired comparisons of TLS density between pre‐invasive and invasive regions specifically within MIA lesions and comparisons across synchronous tumors from the same patients. These analyses provide complementary evidence linking TLS density to pathological invasion within individual lesions and patients.

TLSs are widely recognized as ectopic sites of antigen presentation and immune activation whose presence correlates with favorable prognosis and immunotherapy response across multiple solid tumors [38, 39, 40, 58, 59]. However, accumulating evidence indicates that TLSs are heterogeneous in maturation state, distinguished by FDC networks, germinal centers, and specialized vasculature. In our GGN cohorts, pathological invasion was associated with a sharp accumulation of TLSs, but these newly formed TLSs were overwhelmingly immature (iTLSs), whereas the number of mature TLSs (mTLSs) remained relatively stable across AIS, MIA, and IAC. Therefore, the human immune system seems to respond vigorously to emerging lung malignancy by assembling TLS‐like aggregates, yet fails to efficiently progress them to fully competent mTLSs capable of sustaining productive anticancer immunity. The predominance of iTLSs may thus represent a suboptimally instructed local response that mirrors the bystander T‐cell compartment and contributes to the paradoxical coexistence of dense immune hubs with ongoing tumor invasion.

These insights have important diagnostic and therapeutic implications. Our findings suggest that specific immune features, including lymphocyte abundance and TLS density, may be inferable from radiologic examinations to improve noninvasive risk stratification of subsolid nodules. For instance, incorporating certain radiographic parameters that correlate with a high TLS burden would help flag suspicious lesions that have crossed or are poised to cross the invasive boundary. In addition, our data provide experimental support for immunomodulatory approaches that actively remodel the TLS compartment in early disease. Pharmacologic augmentation of LTβR signaling or relief of checkpoint‐mediated immunosuppression holds promise for converting iTLS‐dominated niches into mTLS‐enriched milieus while reducing tumor progression. These results align with broader efforts to establish TLSs as prognostic and predictive biomarkers in lung cancer and other malignancies, and also raise the possibility that carefully timed immune‐interception strategies could be deployed in selected high‐risk GGNs to delay or even reverse pathological invasion.

### 3.1 Limitations of the Study

Several limitations of our study need to be acknowledged. First, the human cohorts are retrospectively assembled from surgically resectable GGNs and comprise a modest number of AIS/MIA cases compared with IAC. Second, the small sample size limits our ability to distinguish histology‐associated immune differences from potential effects of sex, age, smoking history, and oncogenic genotype. Third, the analyses are largely cross‐sectional along the histopathological continuum and do not capture true longitudinal evolution within individual nodules, hindering causal inference about invasive transition and TLS emergence. Fourth, TLS identification and staging rely on histomorphology and a restricted mIHC marker panel without definitive functional annotation. Fifth, the murine model is based on intrapulmonary injection of a single cell line, which does not fully recapitulate the natural history and immunologic context of human GGNs. Finally, while we implicate LTβR ligands and PD‐1 blockade in promoting TLS maturation and tumor control, these interventions have broader immunological effects. Our experiments therefore do not establish whether TLS maturation mediates the antitumor effects, which requires selective perturbation of TLS development or function.

## 4 Conclusions

Our work highlights the potential value of incorporating detailed immune profiling and TLS phenotyping into the understanding and management of early lung adenocarcinoma. At the mechanistic level, we provide a basis for future studies that combine spatial transcriptomics, single‐cell BCR/TCR sequencing, and functional perturbations to dissect antigen circuits and cellular interactions within TLSs at the invasive front. From a translational perspective, TLS metrics could potentially be evaluated using medical imaging and complement radiologic features to refine risk models for GGNs and inform decisions regarding surveillance vs. intervention. Building on this rationale, prospective clinical trials that integrate TLS‐oriented biomarkers and therapeutic strategies will be essential to determine whether deliberate promotion of TLS maturation can be safely harnessed to intercept lung cancer at its earliest detectable stages.

## 5 Methods

### 5.1 Patient Cohorts and Human Specimens

The study was approved by the Ethics Committee of Ren Ji Hospital (approval no. RA‐2021‐515) and conducted in compliance with the Declaration of Helsinki and Good Clinical Practice guidelines. Written informed consent was obtained from all enrolled participants. Five partially overlapping cohorts of patients with lung ground‐glass opacity nodules (GGNs) at Ren Ji Hospital were analyzed. (i) The single‐cell RNA sequencing (scRNA‐seq) cohort comprised 12 fresh tumor specimens from 11 patients (Table S1); (ii) The TCRβ sequencing cohort comprised 27 fresh tumor specimens, 24 matched lung tissues, and 24 peripheral blood mononuclear cell (PBMC) samples from 24 patients (Table S7); (iii) The whole‐slide immunohistochemistry (IHC) cohort comprised 51 formalin‐fixed and paraffin‐embedded (FFPE) tumor specimens from 51 patients (Table S5); (iv) The tissue microarray (TMA) cohort comprised 177 FFPE tumor specimens from 177 patients and was subjected to multiplex immunohistochemistry (mIHC) analysis (Table S12); (v) The histopathological TLS assessment cohort comprised 265 FFPE tumor specimens from 242 patients (Table S8).

### 5.2 Sample Collection and Processing

Fresh tumor specimens, lung tissues, and blood samples were collected at the time of surgery. PBMCs were isolated promptly after blood collection and stored at ‐80°C. Tumor specimens allocated to scRNA‐seq were processed immediately to generate single‐cell suspensions. Samples allocated to TCRβ sequencing were preserved in liquid nitrogen before DNA extraction. Tissues allocated to histopathological assessment, IHC, or mIHC were fixed in formalin and embedded in paraffin.

### 5.3 Single‐Cell RNA Sequencing

Fresh tumor specimens were cut into approximately 1 mm^3^ pieces in RPMI‐1640 medium (Gibco) supplemented with 10% fetal bovine serum, and enzymatically digested with Liberase TL (0.1 mg/mL, Roche) and DNase I (0.02 mg/mL, Roche) for 30 min on a rotor at 37°C. The resulting cell suspension was filtered through a 70 µm Cell‐Strainer (BD Biosciences) and centrifuged at 400 × g for 5 min at 4°C. Cell pellets were resuspended in red blood cell lysis buffer (Invitrogen), incubated on ice for 3 min, and washed twice with PBS. Single‐cell suspensions were adjusted to 300 cells/µL, and approximately 7,000‐10,000 cells were loaded per chip channel. Libraries were prepared using the Chromium Single Cell 3′ Library, Gel Bead & Multiplex Kit and Chip Kit with V2 chemistry (10× Genomics) according to the manufacturer's instructions. Purified libraries were sequenced on an Illumina NovaSeq 6000 with 150‐bp paired‐end reads. For quality control, the unique molecular identifier (UMI) count matrix of each sample was generated using Cell Ranger v1.3.1. We removed mitochondrial genes, immunoglobulin genes, high‐abundance lncRNAs, and genes linked with poorly supported transcriptional models [60]. Genes detected in fewer than three cells across the dataset were also excluded. Cells were excluded if they had < 500 or > 7,500 UMIs, < 200 or > 3,000 expressed genes, or > 20% mitochondrial reads. After quality control, 46,377 cells were retained for downstream analyses.

### 5.4 Data Normalization and Clustering

Single‐cell data were analyzed using Seurat v2.3.4 [61]. For each sample, we normalized the UMI count matrix by using the global‐scaling normalization method “LogNormalize” implemented in the Seurat function NormalizeData. We next used the FindVariableGenes function to detect variable genes for downstream principal component analysis (PCA). To avoid interference, gene features associated with the cell cycle, type I interferon (IFN) response, and general cell stress were excluded from the variable gene list [60]. To reduce the dimensionality of this dataset, the resulting variable genes were scaled and subjected to PCA, and the top significant principal components (PCs) were further summarized by t‐distributed stochastic neighbor embedding (t‐SNE) using the RunTSNE function. We applied the modularity optimization algorithm to iteratively group cells together by using the FindClusters function. To combine multiple samples, we performed batch correction with ComBat at the default settings [62]. The batch‐corrected dataset was then converted to a Seurat object and analyzed as above. Marker genes for each cell cluster were found using the FindAllMarkers function. For individual samples, differential gene expression analysis was performed between cells in each cluster and all cells in the other clusters using a Wilcoxon rank‐sum test. In the combined data of multiple samples, differentially expressed genes for each cluster were found using a likelihood‐ratio test. Cell clusters were annotated using canonical marker genes.

### 5.5 Identification of Cell Subset Marker Genes

We defined cell subset marker genes following a previous study [63]. A gene j was considered a marker gene for cell subset ci if it met all of the following criteria: (1) j was detected in ≥ 3 cells with at least 3 counts within ci; (2) j was expressed significantly higher in ci compared to the complement subsets, assessed by a two‐tailed Mann–Whitney U test with Benjamini‐Hochberg correction (FDR < 5%); (3) j had the maximal average expression in ci across all subsets; (4) j satisfied ${\mathrm{stepratio}}_{j}^{1}$ > 1.1, where ${\mathrm{stepratio}}_{j}^{1}$ was the ratio of the highest to the second‐highest subset average expression for gene j after adding a pseudovalue of 0.1. For the eight major cell types, cell‐type specific signatures were defined as the top 20 marker genes ranked by descending ${\mathrm{stepratio}}_{j}^{1}$ for each cell type.

### 5.6 TCR Sequencing and Analysis

TCR β‐chain sequencing was performed by utilizing genomic DNA extracted from fresh tumor specimens, lung tissues and PBMC samples. After preprocessing the raw sequencing data for each sample, we calculated the number of unique clones (N) and the total count of TCR sequences $U\;=\sum_{i=1}^{N}u_{i}$, where u_i_ was the read count of the clonotype i=1,2…,N. The proportion of each clonotype was defined as $p_{i}=\frac{u_{i}}{U}$. Diversity of clonotypes (D) was calculated by the normalized Shannon entropy, given by $D\;=-\sum_{i\;=1}^{N}\frac{p_{i}\times \mathrm{lo}g_{2}\left(p_{i}\right)}{\mathrm{lo}g_{2}\left(N\right)}$. The clonality index (CI) was defined as the complement of diversity, i.e., CI  =  1 − D. Relative clonotype richness (R) was calculated as the average unique clonotypes per cell (assuming one unique clonotype per cell), given by $R\;=\frac{N}{U}$. To assess the prevalence of shared clonotypes across samples within the same patient, we measured the overlap between T‐cell repertoires using the Jaccard index and Horn index. The Jaccard index was defined as $J_{\mathrm{AB}}=\frac{N_{A}\cap N_{B}}{N_{A}\cup N_{B}}$, where N_A_ and N_B_ were the numbers of unique clones in samples A and B, respectively. The Horn index was defined as $H_{\mathrm{AB}}=\frac{2\sum_{i=1}^{N}p_{\mathrm{Ai}}\times p_{\mathrm{Bi}}}{\sum_{i=1}^{N}{\left(p_{\mathrm{Ai}}\right)}^{2}+\sum_{i=1}^{N}{\left(p_{\mathrm{Bi}}\right)}^{2}}$, where p_Ai_ and p_Bi_ were the proportions of the shared clonotype i in samples A and B, respectively. J and H ranged from 0 to 1 with increasing values indicating more shared clonotypes. The frequency distribution of TCR abundance was fitted to a discrete power‐law distribution of the form f_(k)_ =  Ck^−α^, where $C\;=\frac{1}{\zeta_{\left(\alpha \right)}}$, ζ denoted the Riemann zeta function and f_(k)_ was the frequency of TCRs detected k times, by maximum likelihood estimation as described in a previous study [64]. We identified viral T‐cell motifs as previously described [65]. Briefly, the grouping of lymphocyte interactions by paratope hotspots (GLIPH) was used to cluster CDR3 rearrangements and identify T‐cell specificity groups [66]. The CDR3 sequences of the TCR β‐chain from this study were used in conjunction with publicly available, tetramer‐defined viral CDR3 sequences [67]. Viral motifs were defined as GLIPH motifs containing ≥ 3 viral tetramer‐derived CDR3 sequences and showing enrichment for a given V‐gene (P  <  0.05 by Fisher's exact test).

### 5.7 Orthotopic Murine Lung Adenocarcinoma Model

All animal experiments were approved by the Institutional Animal Care and Use Committee of Shanghai Jiao Tong University (approval no. A2024240‐001). Mice were housed under pathogen‐free conditions at the Laboratory Animal Center of Shanghai Jiao Tong University. An orthotopic murine lung adenocarcinoma model was established by intrapulmonary injection of tumor cells. In detail, 6 to 8‐week‐old female C57BL/6 mice were randomized and anesthetized with 2% isoflurane and immobilized in a stereotaxic frame on a heated surface in the right lateral decubitus position. The left chest wall was subjected to a 1‐cm incision between the seventh and ninth intercostal spaces, followed by blunt dissection until visualization of the parietal pleura. A total of 2 × 10^5^ 889‐S1 cells, 889‐S1‐EV (empty vector) cells, 889‐S1‐LIGHT cells, or 889‐S1‐LTαβ cells were orthotopically injected into the left lung with a Hamilton syringe at a depth of 1.5‐3 mm (5 mice per group). For temporal characterization of tumor growth, micro‐CT images were acquired using an IRIS imaging system (Inviscan). For the anti‐PD‐1 treatment experiment, intraperitoneal injection of anti‐mouse PD‐1 antibody or matched isotype control (10 mg/kg, Selleck) was administered on days 7 and 10 after tumor implantation. On day 14, mice in the treatment cohorts were euthanized. Intrapulmonary tumor tissues were collected, fixed in formalin, and processed for subsequent pathological evaluation and mIHC analysis. Pathological evaluation and mIHC analysis were performed with investigators blinded to group allocation, and no mice or data points were excluded. Quantitative PCR (qPCR) was performed to verify the overexpression of LIGHT or LTαβ in 889‐S1 cells using the following primers: LIGHT‐F: 5′‐AGCAGCACATCTTACAGGAGC‐3′; LIGHT‐R: 5′‐AGCTGCACTTTGGAGTACACA‐3′; LTα‐F: 5′‐CACCTCTTGAGGGTGCTTGG‐3′; LTα‐R: 5′‐TGGGGTACCCAACAAGGTG‐3′; LTβ‐F: 5′‐GTTCAACAGCTGCCAAAGGG‐3′; LTβ‐R: 5′‐CATCCAAGCGCCTATGAGGT‐3′.

### 5.8 Immunohistochemical Staining

Immunohistochemistry (IHC) was performed on 5 µm‐thick FFPE tissue sections. Slides were baked, deparaffinized in xylene, rehydrated through graded alcohols, and antigen retrieved with 10 mM citrate buffer (pH 6.0) in a steam pressure cooker. Preprocessed tissues were treated with peroxidase block (Dako) to quench endogenous peroxidase activity, blocked using protein block (Dako), and incubated with primary antibodies. Slides were then washed in 50 mM Tris‐Cl (pH 7.4) and incubated with horseradish peroxidase‐conjugated secondary antibodies. Immunoperoxidase staining was developed using 3,3′‐diaminobenzidine (DAB) chromogen. Slides were counterstained with hematoxylin, dehydrated through graded alcohol and xylene, and coverslipped using mounting solution. Primary antibodies against CD3 (Kit‐0003), CD4 (RMA‐0620), CD8 (RMA‐0514), CD20 (Kit‐0001), CD56 (Kit‐0028), and CD68 (Kit‐0026) were purchased from MXB Biotechnologies. Primary antibodies against mast cell tryptase (ab134932), PD‐1 (ab52587), and PD‐L1 (ab205921) were purchased from Abcam.

### 5.9 Multiplex Immunohistochemistry

Multiplex immunohistochemistry (mIHC) was performed using the Opal Multiplex IHC kit (Akoya Biosciences, NEL861001KT) according to the manufacturer's instructions. In brief, antigen was retrieved using AR6 and AR9 buffer followed by 10 min of blocking before incubation with primary antibodies for 30 min at room temperature. Horseradish peroxidase‐conjugated secondary antibodies were added and incubated for 10 min. Signals were developed using tyramide signal amplification (TSA). Finally, slides were counterstained with DAPI and mounted with antifade mountant. Multispectral images were collected using the Vectra Polaris multispectral imaging system (Akoya Biosciences) at 20× magnification. The following human primary antibodies were used: CD20 (#48750, Cell Signaling Technology), CD4 (#48274, Cell Signaling Technology), CD8 (#85336, Cell Signaling Technology), CD21 (ab227662, Abcam), and pan‐cytokeratin (Kit‐0009, MXB Biotechnologies). The following mouse primary antibodies were used: CD20 (#70168, Cell Signaling Technology), CD4 (#25229, Cell Signaling Technology), CD8 (#98941, Cell Signaling Technology), CD21 (ab227662, Abcam), and tdTomato (#20163, Cell Signaling Technology).

### 5.10 TLS Identification and Quantification

TLSs were identified on H&E‐stained whole‐slide images by a board‐certified pathologist as discrete, densely packed lymphocyte aggregates with discernible, smooth contours. TLS density was calculated as the number of TLSs within an analyzed region divided by the area of that same region and expressed as TLSs/mm^2^. TLS structural maturation status was evaluated by mIHC according to the presence of a CD21^+^ follicular dendritic cell network within a lymphoid aggregate. TLSs lacking such a network were designated immature TLSs (iTLSs), whereas those containing a discernible CD21^+^ follicular dendritic cell network were designated mature TLSs (mTLSs). All images were analyzed using QuPath v0.7.0.

### 5.11 Statistical Analysis

Statistical analyses were conducted using R v3.5.0 and GraphPad Prism v6.00. TCGA datasets were obtained from UCSC Xena and processed as previously described [68]. Gene signature scores were calculated by single‐sample gene set enrichment analysis (ssGSEA) using the R package GSVA [69]. Survival analysis was conducted with the R package ‘survival’. Survival curves were estimated using the Kaplan–Meier method and compared using the log‐rank test. Independent groups were compared using Mann–Whitney U tests, with exact *p* values for small‐sample comparisons where applicable and asymptotic *p* values for larger samples. Paired continuous variables were compared using paired t tests or Wilcoxon signed‐rank tests, according to the distribution of within‐pair differences. Paired t tests require approximately normally distributed differences, whereas Wilcoxon signed‐rank tests assume approximately symmetric differences. For paired TLS measurements with markedly asymmetric differences, an exact sign test was used, excluding zero differences from the test. IHC effect sizes were expressed as mean paired differences with 95% confidence intervals (CIs). TLS effect sizes were expressed as differences between group medians for independent comparisons or medians of within‐pair differences for paired comparisons. The 95% CIs were estimated using the percentile bootstrap method with 100,000 resamples. The Holm method was used to control the family‐wise error rate within related sets of comparisons, whereas the Benjamini–Hochberg method was used to control the false discovery rate in exploratory analyses involving multiple cell types or genes. Associations between variables were evaluated using Pearson's correlation coefficient or Spearman's rank correlation coefficient. Categorical variables were compared using two‐sided Fisher's exact tests. Individual lesions were treated as independent observations without adjustment for within‐patient clustering. Matched tissue and region comparisons retained their pairing, and TCR repertoire comparisons used patient‐level summaries. Sample sizes, statistical tests, and multiple‐testing correction are specified in the corresponding figure legends. All tests were two‐sided, and p < 0.05 was considered statistically significant, using adjusted *p* values where multiple‐testing correction was applied.

## Author Contributions

Y.F., G.Z. and P.M. conceptualized and designed the study; C.H., M‐C.C., J.W., H.L., S.Z., Z.Z., Q.Z., Y.C., J.Z., K.Y., Y.S., C.C., H.L., Y.F., Z.Y. and X.Z. acquired, analyzed and interpreted the data; Y.F., G.Z., P.M. and M‐C.C. drafted the manuscript. All authors read and approved the final version of the manuscript.

## Funding

This work was supported by the Noncommunicable Chronic Diseases‐National Science and Technology Major Project (2026ZD0557200), National Key R&D Program of China (2024YFA0916700), National Natural Science Foundation of China (82373351; 82573385; 82273273; 82673853; 82303030; 82503860), innovative research team of high‐level local universities in Shanghai (SHSMU‐ZLCX20210200), Science and Technology Commission of Shanghai Municipality (23ZR1438800), Guizhou Provincial Science and Technology Projects (grant number Qiankeherencai XKBF [2025]024), and Shanghai Sailing Program (23YF1441100).

## Conflicts of Interest

The authors declare no conflicts of interest.

## Supporting information




**Supporting File 1**: advs78670‐sup‐0001‐SuppMat.pdf.

**Supporting File 2**: advs78670‐sup‐0002‐SuppMat.xlsx.

## Data Availability

The raw sequencing data have been deposited in NCBI SRA database (https://www.ncbi.nlm.nih.gov/sra/) under the accession number SRP262066. The processed data have been deposited in NCBI GEO database (https://www.ncbi.nlm.nih.gov/geo/) under the accession number GSE150938. In addition, we have deposited the analytical workflow on GitHub (https://github.com/zhuanglab‐rj/LC_scRNA_TLS).
