# Supplementary material for: Single‐Cell Map of Early Lung Adenocarcinoma Uncovers Invasion‐Associated Tertiary Lymphoid Structures
Source: Adv Sci (Weinh). 2026 Nov 9:e78670. Online ahead of print. doi: 10.1002/advs.78670 (PMC13748784; doi:10.1002/advs.78670)
Supplement: Supplementary file 1 [file ADVS-9999-e78670-s002.pdf]

Supplementary Figure 1.

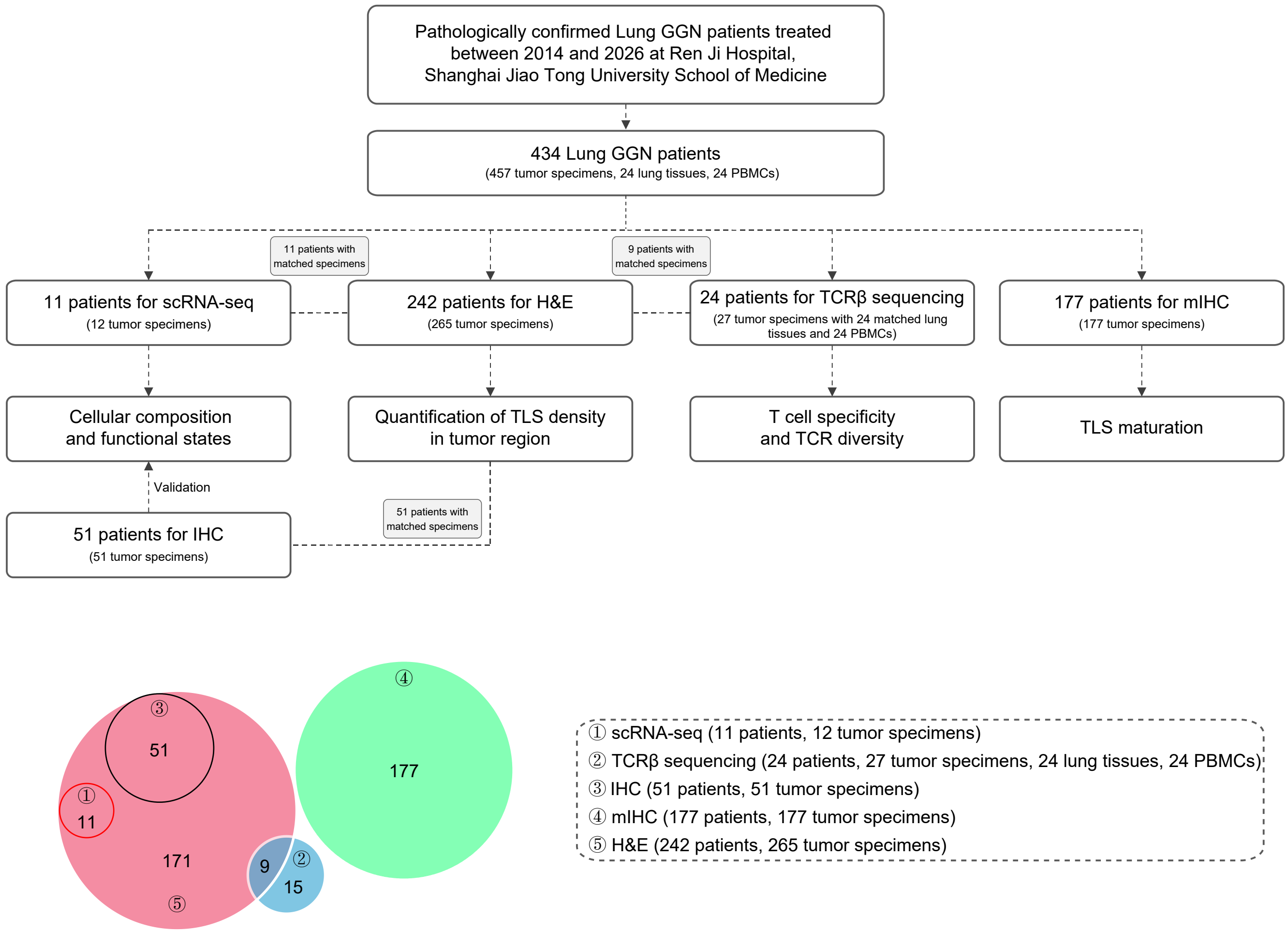

Supplementary Figure 1. Study flow diagram and Venn diagram illustrating the composition and overlap of the lung GGN cohorts. The overall cohort comprised 434 patients with pathologically confirmed lung GGN, including 457 tumor specimens, 24 lung tissues, and 24 PBMCs. 12 tumor specimens underwent scRNA-seq, 51 patients underwent IHC, 177 patients underwent mIHC, and 24 patients including 27 tumor specimens, 24 lung tissues, and 24 PBMCs underwent TCRβ sequencing. Finally, 265 FFPE tumor specimens from 242 patients underwent H&E staining, which included scRNA-seq cohort, IHC cohort, and part of TCRβ sequencing cohort.

Supplementary Figure 2.

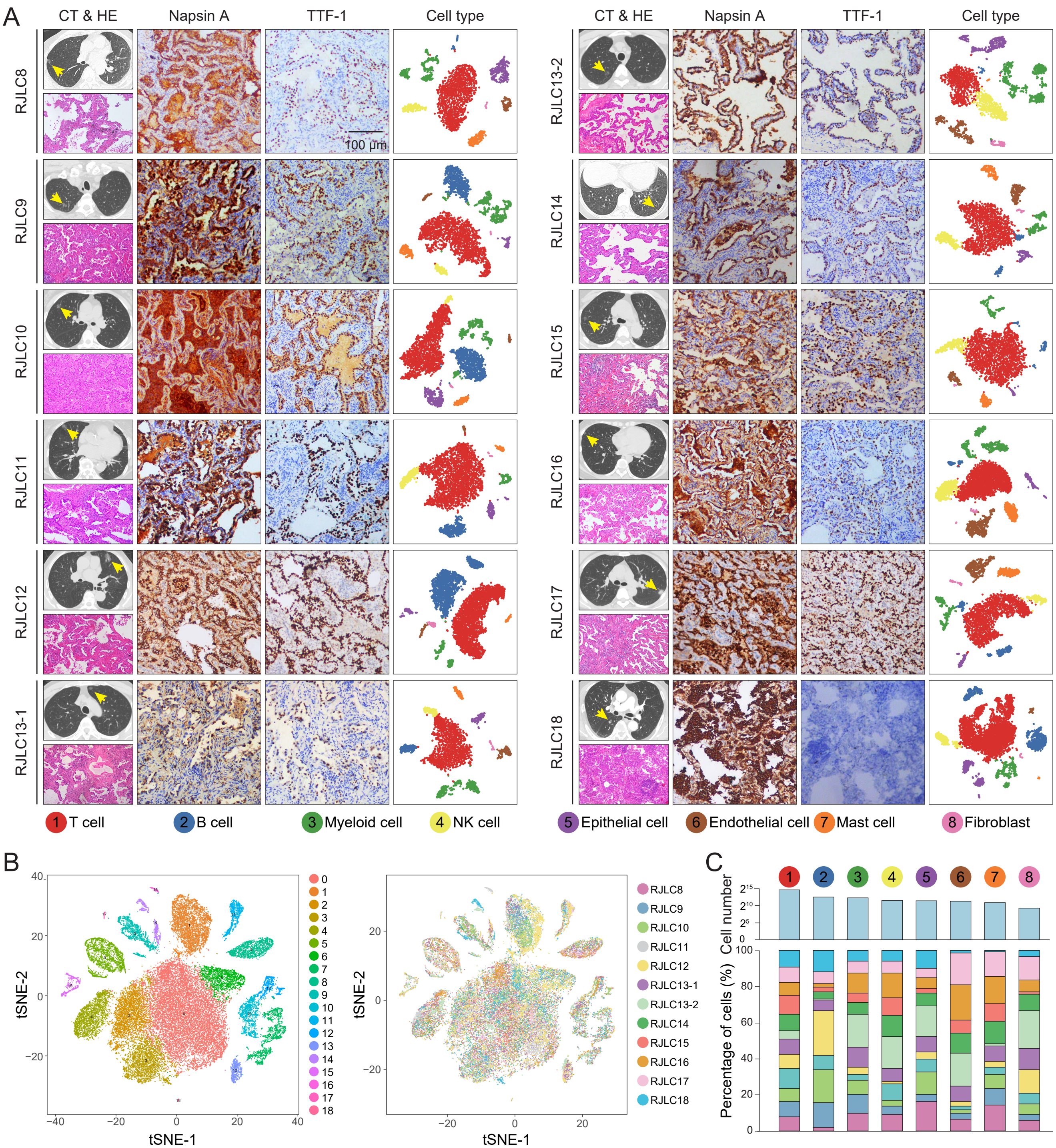

Supplementary Figure 2. (A) CT image, H&E, IHC staining for Napsin A and TTF-1, and t-SNE plots showing the 8 major cell types identified by scRNA-seq analysis in each human lung GGN. (B) t-SNE plots showing the 19 clusters identified by scRNA-seq analysis (left) and patient of origin (right) across all cells from 12 human lung GGNs. (C) Stacked bar graph showing the fraction of cells from each of 12 human lung GGNs for the 8 major cell types.

Supplementary Figure 3.

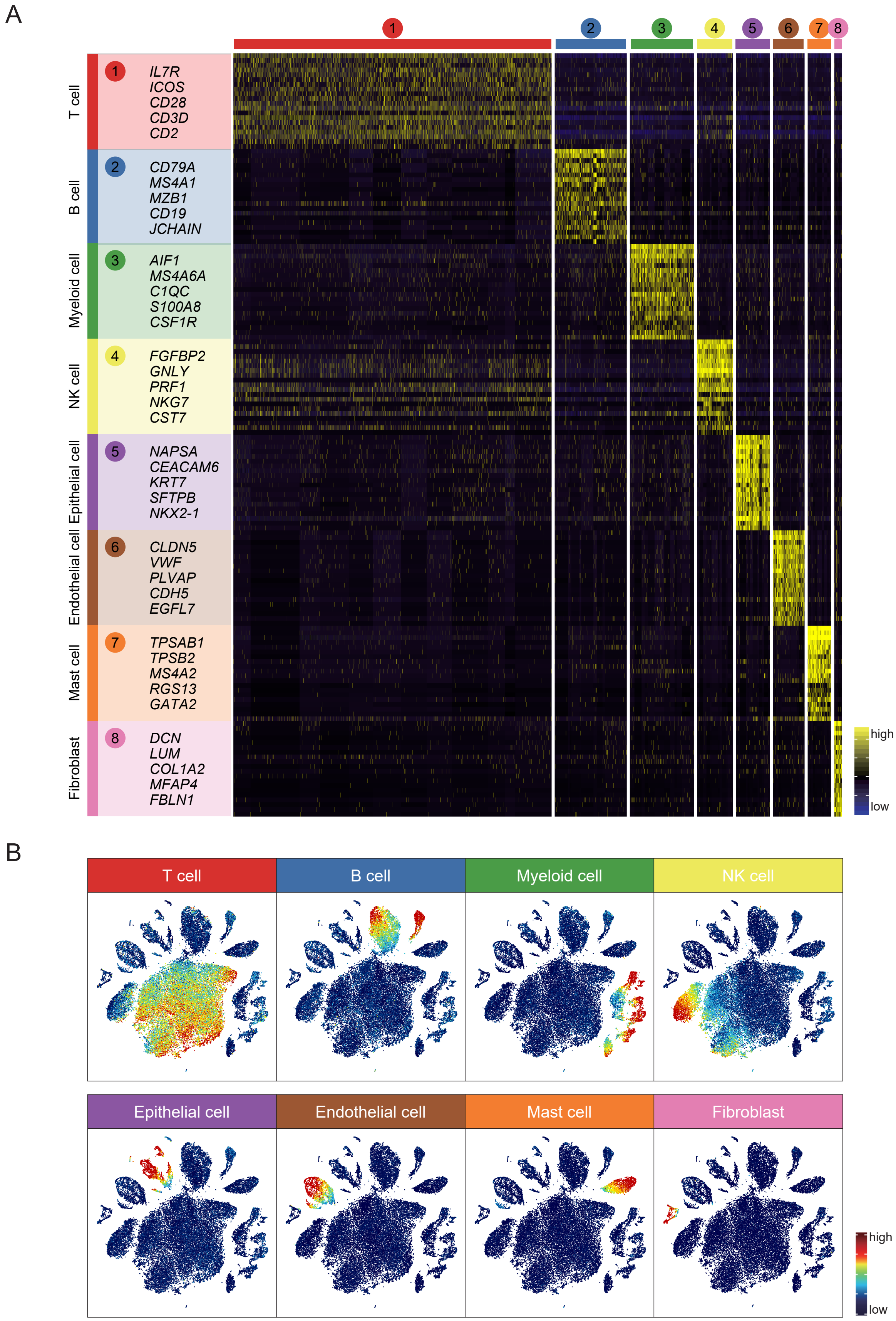

Supplementary Figure 3. (A) Heatmap showing gene expression of selected markers for the 8 major cell types identified by scRNA-seq analysis. (B) t-SNE plots showing signature scores for the 8 major cell types.

Supplementary Figure 4.

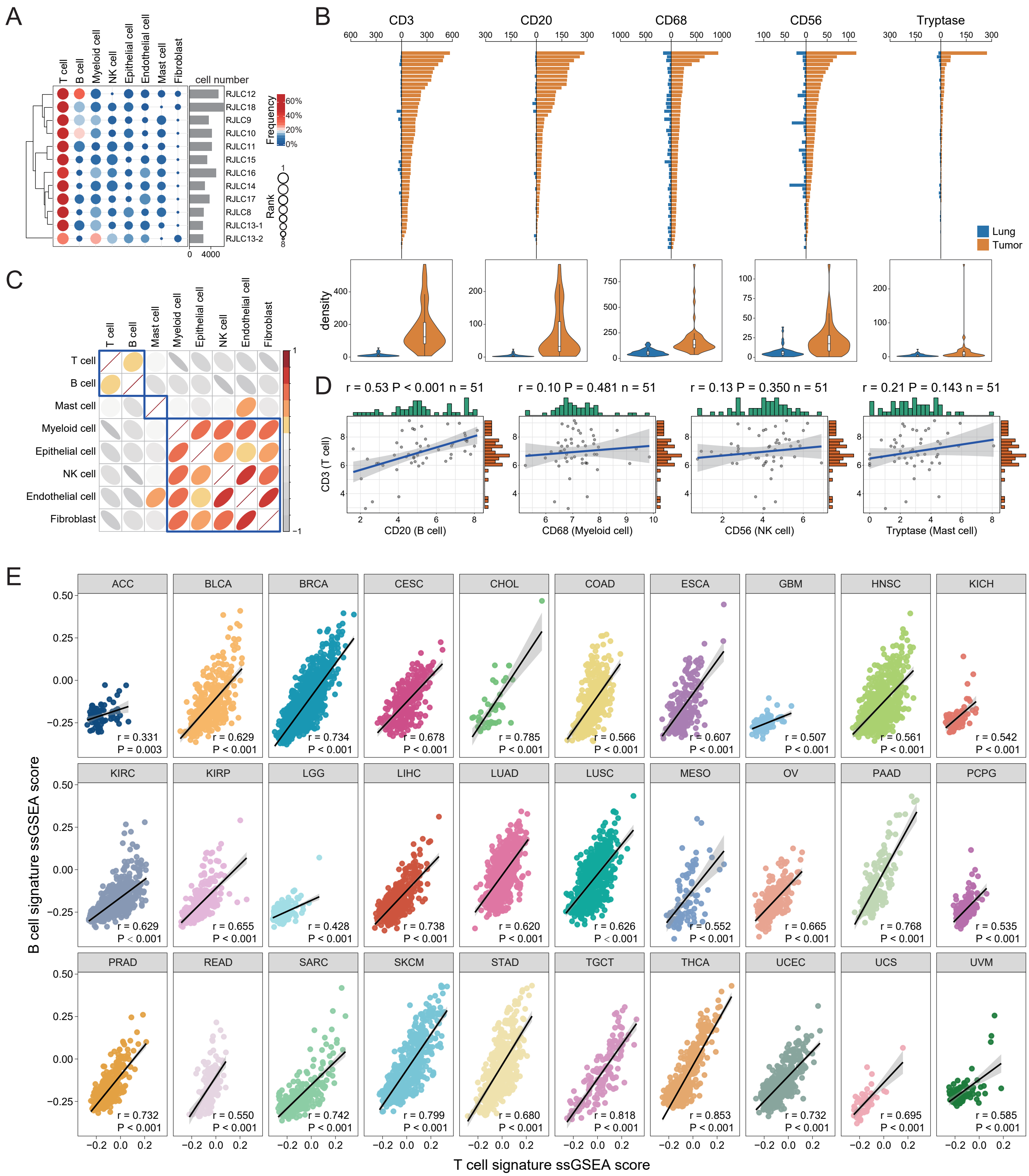

Supplementary Figure 4. (A) Dot plot showing the distribution of 8 major cell types identified by scRNA-seq analysis in each human lung GGN. (B) Quantification of CD3+, CD20+, CD68+, CD56+, and tryptase+ cell densities with IHC analysis in tumor lesions and paired normal tissues from a cohort of 51 human lung GGNs. (C) Pairwise Spearman correlation analysis of 8 major cell types identified by scRNA-seq analysis in 12 human lung GGNs. (D) Pearson correlation analysis between T cells and B cells, myeloid cells, NK cells, or mast cells in tumor lesions from a cohort of 51 human lung GGNs. (E) Pearson correlation analysis between T cell and B cell ssGSEA scores across 30 TCGA cancer types.

Supplementary Figure 5.

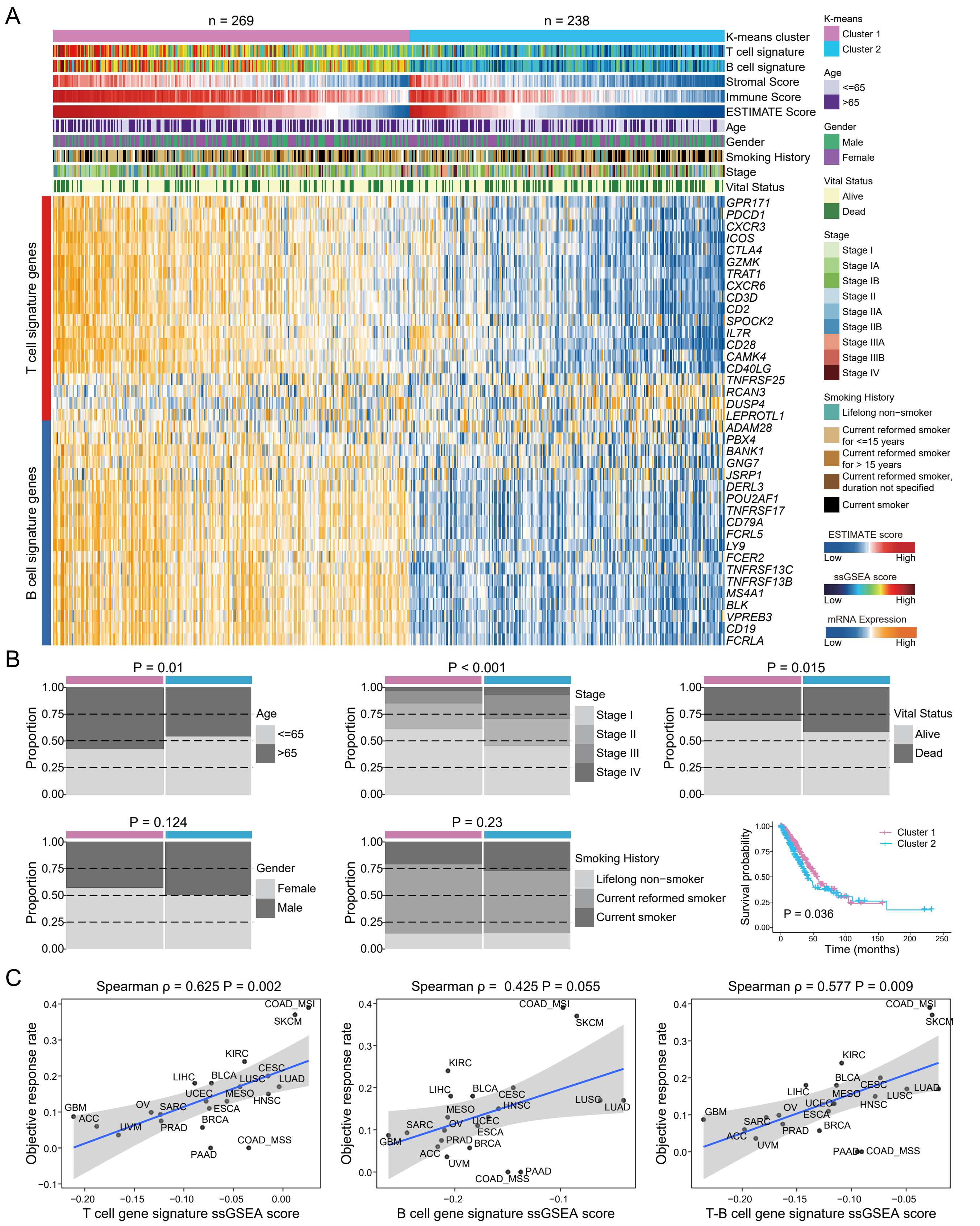

Supplementary Figure 5. (A) Heatmap showing the expression of T cell and B cell signature genes in two TCGA lung adenocarcinoma clusters. (B) Stacked bar graph showing the distribution of age, stage, vital status, gender, and smoking history in two TCGA lung adenocarcinoma clusters. P values were calculated using Fisher's exact tests. Overall survival was analyzed using Kaplan–Meier estimates and two-sided log-rank test. (C) Spearman correlation estimation between overall response rate to anti-PD-1/PD-L1 therapy and ssGSEA score of T cell, B cell, and T-B cell combined gene signatures across 21 cancer types.

Supplementary Figure 6.

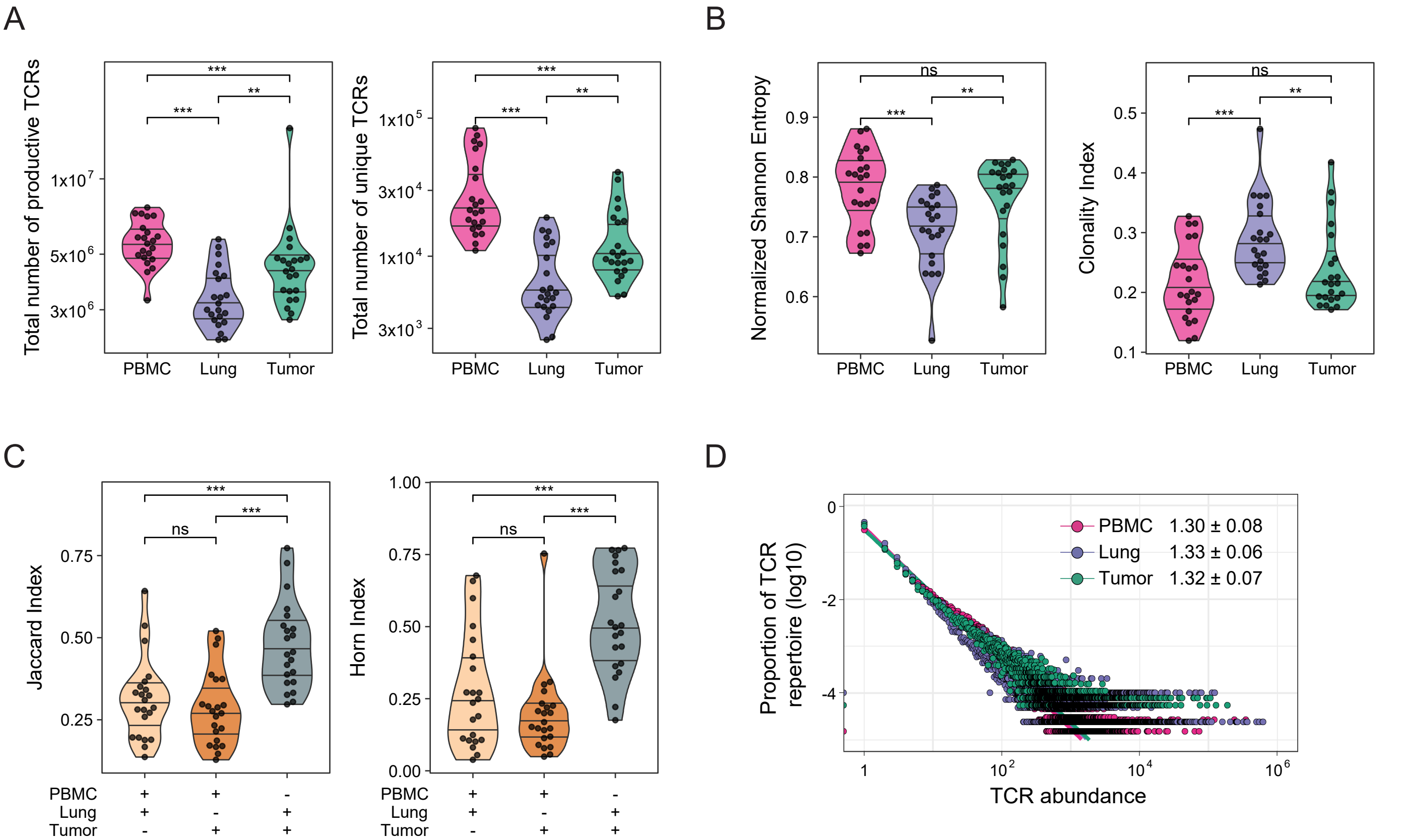

Supplementary Figure 6. (A) Total numbers of productive TCRs and unique TCRs identified in matched PBMC, lung tissues, and tumor samples from a cohort of 22 human lung GGNs. (B) Shannon entropy and clonality index of TCR clonotypes in matched PBMC, lung tissues, and tumor samples from a cohort of 22 human lung GGNs. (C) Jaccard and Horn index of TCR repertoire homology in matched PBMC, lung tissues, and tumor samples from a cohort of 22 human lung GGNs. **P < 0.01, ***P < 0.001, ns = non-significant, Mann-Whitney U test with Holm correction. (D) The frequency distribution of TCR abundance in matched PBMC, lung tissues, and tumor samples was fitted to a discrete power law by maximum likelihood. X-axis represents TCR abundance (size of clone). Y-axis represents the proportion of TCR repertoires (log10). The average power law parameter α of all 22 patients is presented as mean ± SD.

Supplementary Figure 7.

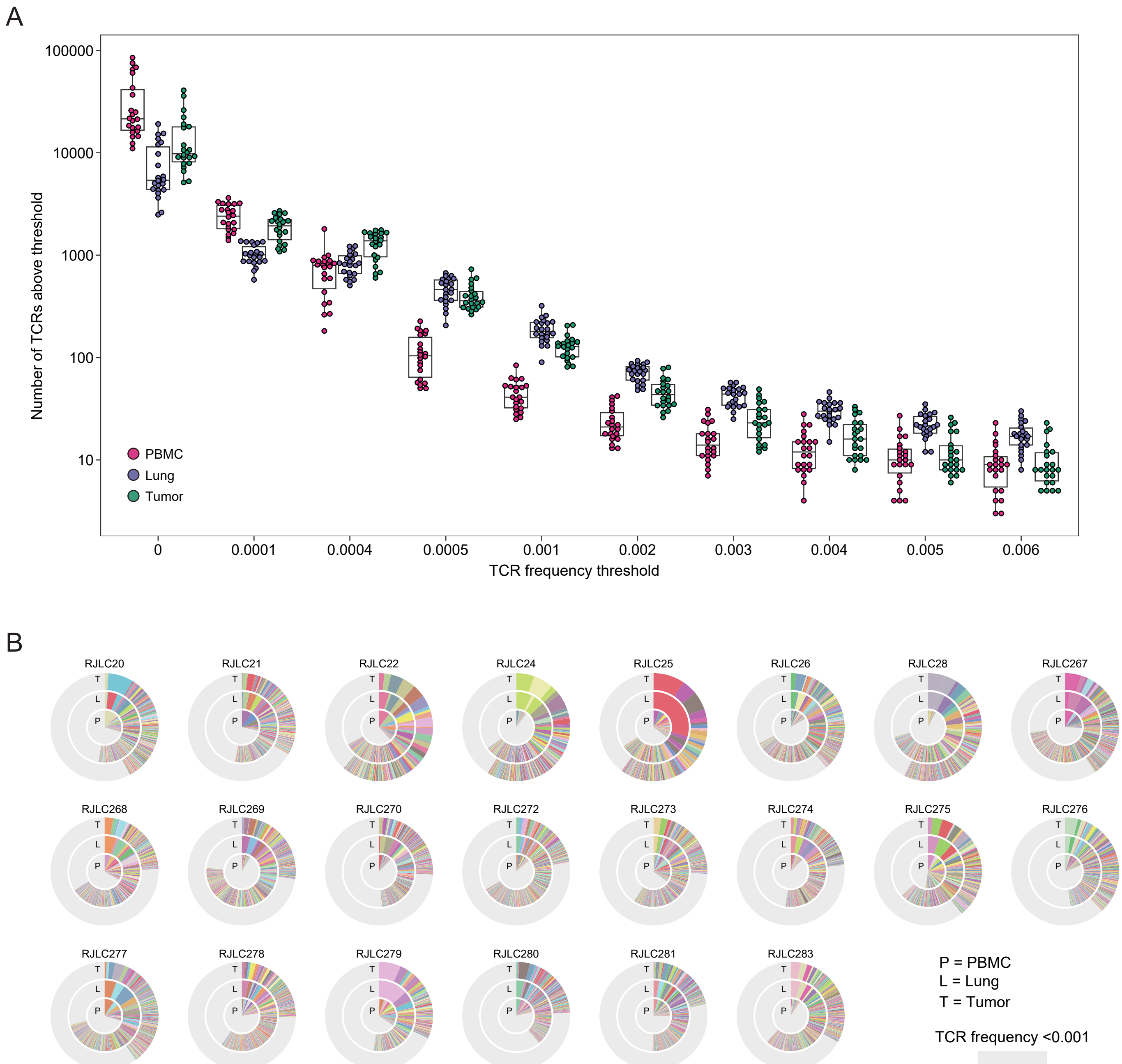

Supplementary Figure 7. (A) Quantification of TCR repertoires detected above a given frequency threshold in matched PBMCs, lung tissues, and tumor samples from a cohort of 22 human lung GGNs. (B) Pie graph showing expanded TCRs (frequency > 0.001) in matched PBMC, lung tissue, and tumor sample of each patient. Each color denotes an expanded TCR.

Supplementary Figure 8.

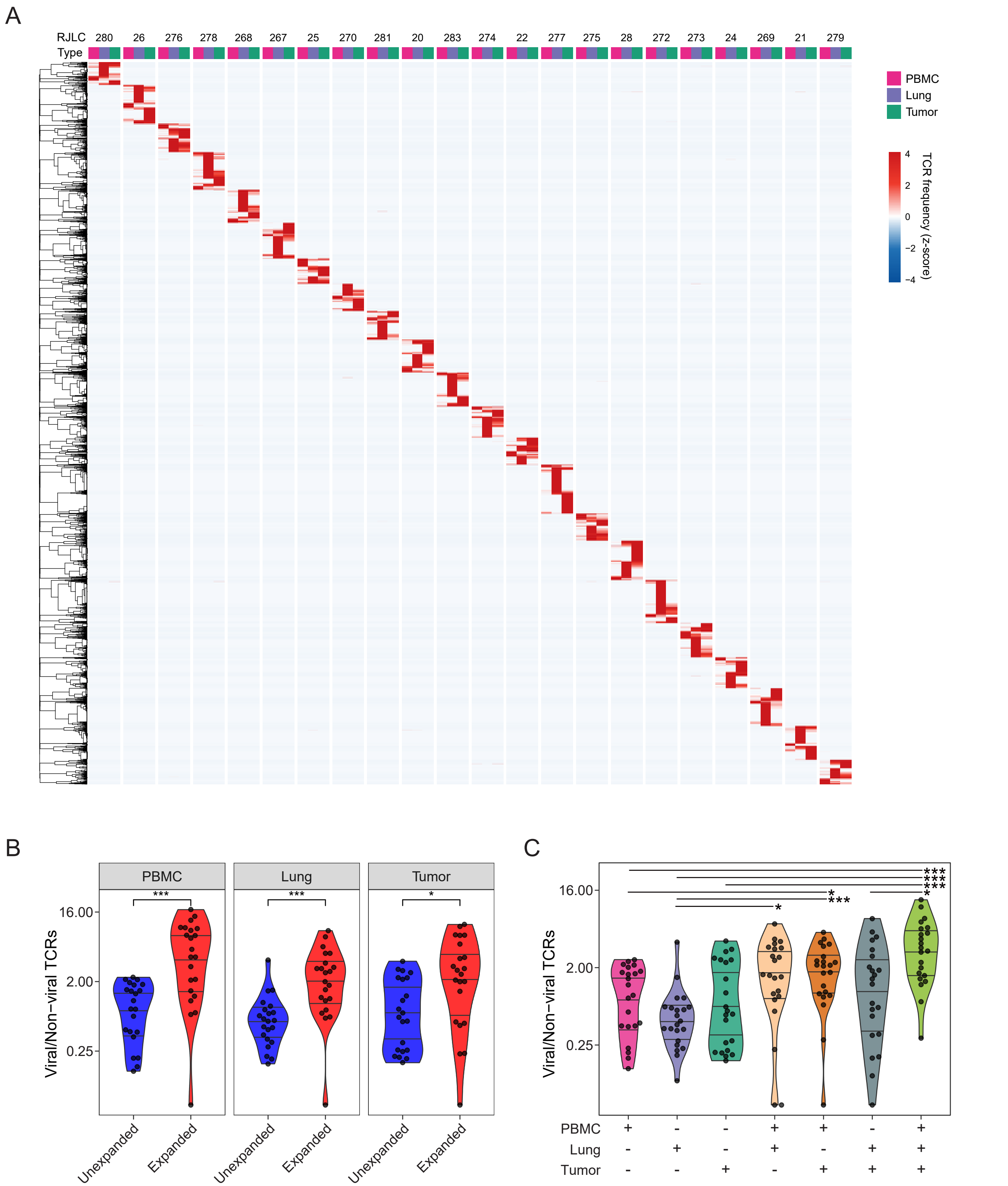

Supplementary Figure 8. (A) Heatmap showing all expanded TCRs (frequency > 0.001) in matched PBMCs, lung tissues, and tumor samples from a cohort of 22 human lung GGNs. (B) Fold difference between viral and non-viral T cell motifs of unexpanded (blue) and expanded (red) TCRs in PBMCs, lung tissues, and tumor samples. *P < 0.05, **P < 0.01, ***P < 0.001, Mann-Whitney U test. (C) Fold difference between viral and non-viral T cell motifs found in PBMCs, lung tissues, and tumor samples as indicated. *P < 0.05, **P < 0.01, ***P < 0.001, Mann-Whitney U test with Holm correction.

Supplementary Figure 9.

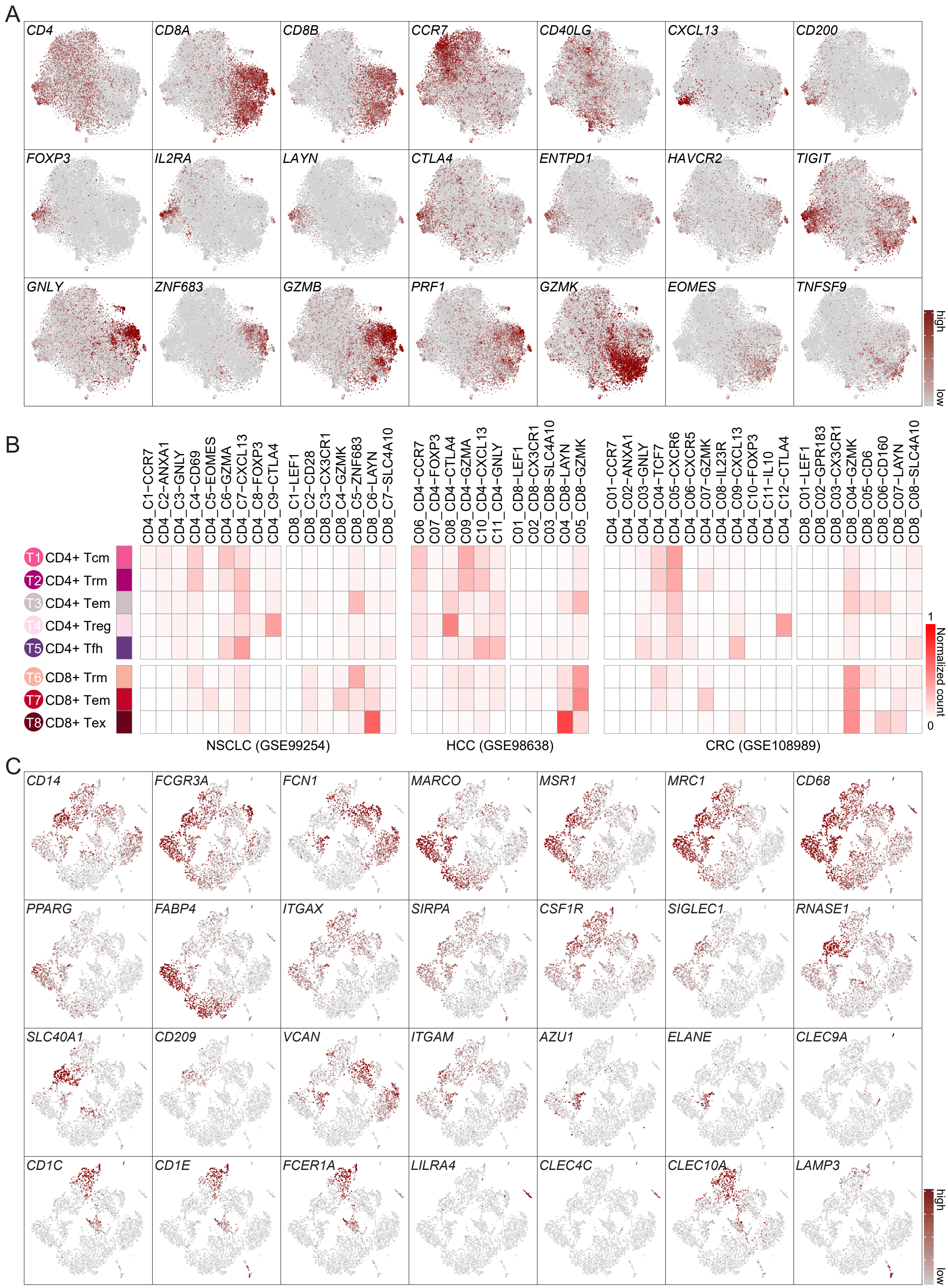

Supplementary Figure 9. (A) t-SNE plots showing gene expression of selected markers for the 8 T cell clusters identified by scRNA-seq analysis. (B) Heatmap showing correlation of the 8 T cell subsets with three published scRNA-seq datasets (GSE99254, GSE98638, and GSE108989). (C) t-SNE plots showing gene expression of selected markers for the 11 myeloid cell clusters identified by scRNA-seq analysis.

Supplementary Figure 10.

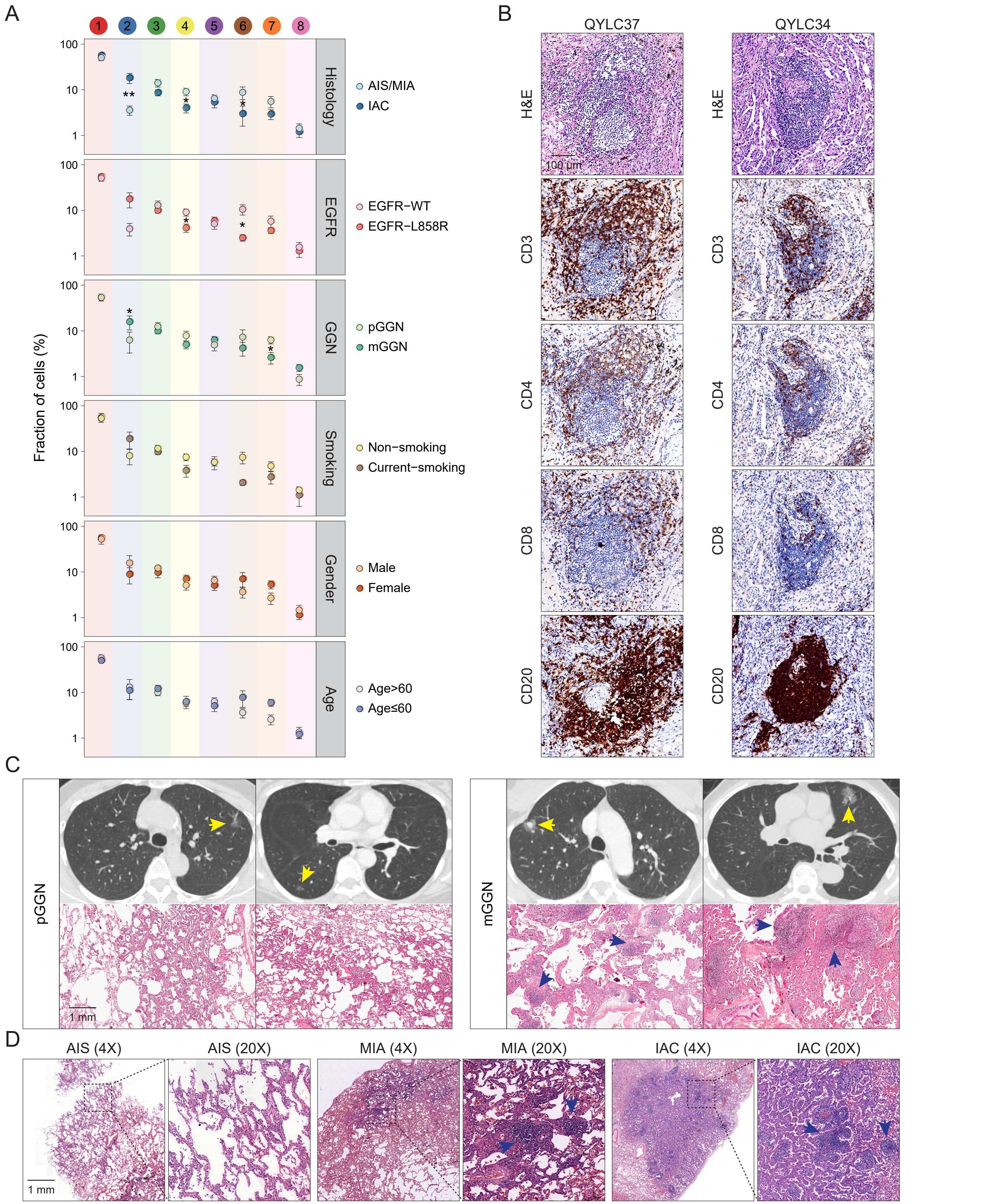

Supplementary Figure 10. (A) Fractions of the 8 major cell types stratified by histology, EGFR alteration, GGN type, smoking history, gender, and age. *P < 0.05, **P < 0.01, Mann-Whitney U test. (B) Representative images of H&E and IHC staining for CD3, CD4, CD8 and CD20 in TLSs of human lung GGNs. Scale bar, 100 μm. (C) Representative CT and H&E images for pGGN and mGGN. Yellow arrows indicate GGNs, and blue arrows indicate TLSs. Scale bar, 1 mm. (D) Representative H&E images for human lung GGNs diagnosed with AIS, MIA and IAC. Blue arrows indicate TLSs. Scale bar, 1 mm.

Supplementary Figure 11.

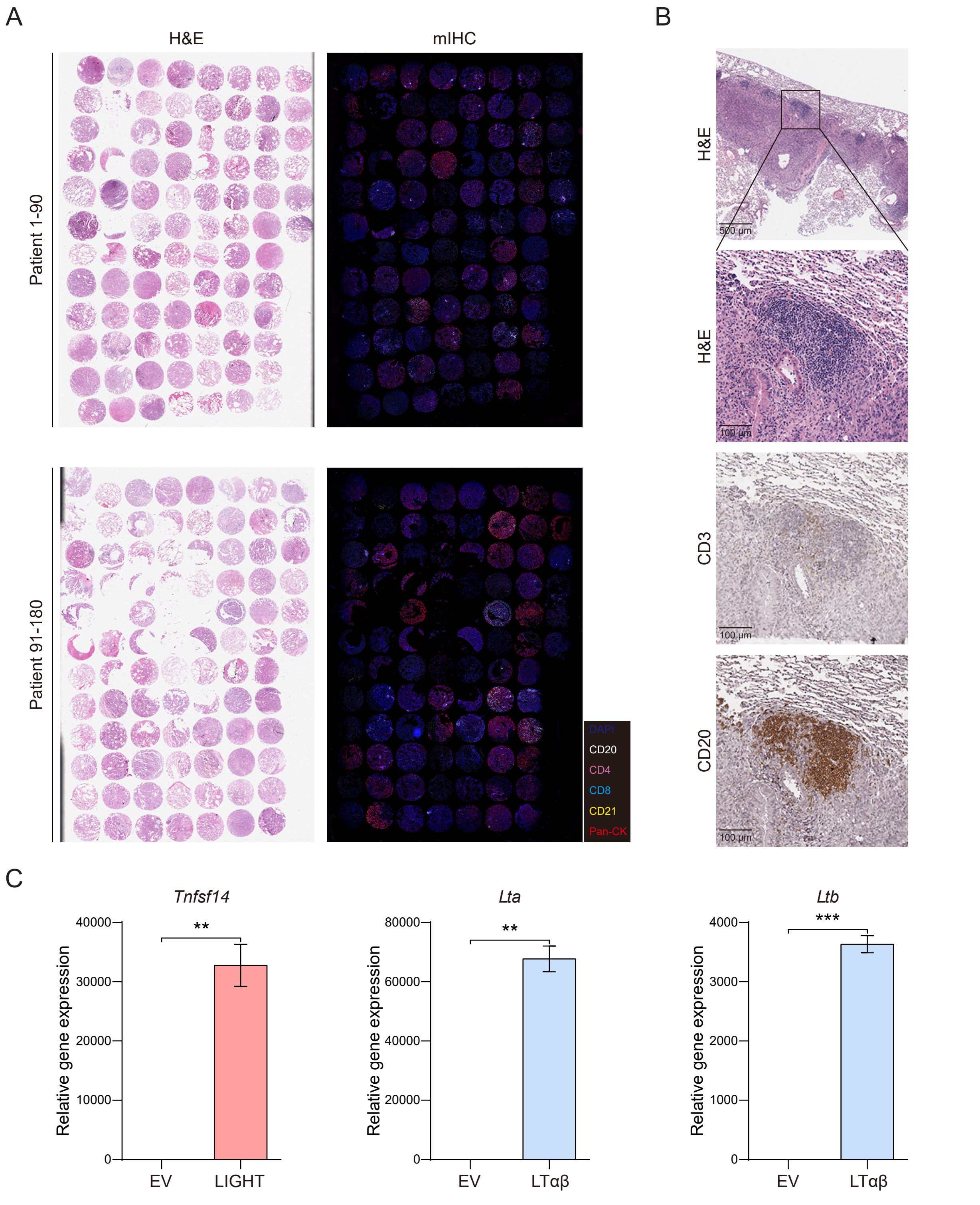

Supplementary Figure 11. (A) H&E and mIHC staining for CD20, CD4, CD8, CD21, Pan-CK, and DAPI in tissue microarrays with 180 human lung GGNs. (B) Representative images of H&E and IHC staining for CD3 and CD20 in TLSs of the orthotopic murine lung adenocarcinoma. (C) Relative gene expression of Tnfsf14, Lta, and Ltb in 889-S1-EV versus 889-S1-LIGHT or 889-S1-LTαβ cells, as assessed by quantitative PCR (qPCR). **P < 0.01, ***P < 0.001, Welch's t-test.
